# Framework for Estimating Indirect Costs in Animal Health Using Time Series Analysis

**DOI:** 10.3389/fvets.2019.00190

**Published:** 2019-06-18

**Authors:** Alyson S. Barratt, Karl M. Rich, Jude I. Eze, Thibaud Porphyre, George J. Gunn, Alistair W. Stott

**Affiliations:** ^1^Department of Rural Economy, Environment and Society, Scotland's Rural College, Faculty of Rural Science and Policy, Edinburgh, United Kingdom; ^2^East and Southeast Asia Regional Office, International Livestock Research Institute, Hanoi, Vietnam; ^3^Epidemiology Research Unit, Department of Veterinary and Animal Science, Northern Faculty, Scotland's Rural College, Inverness, United Kingdom; ^4^Biomathematics and Statistics Scotland, JCMB, The King's Buildings, Edinburgh, United Kingdom; ^5^Royal (Dick) School of Veterinary Studies, The Roslin Institute, University of Edinburgh, Midlothian, United Kingdom

**Keywords:** indirect costs, animal disease, foot and mouth disease, time series modeling, vector error correction model, market impact, disease control strategy

## Abstract

Traditionally, cost-benefit analyses (CBAs) focus on the direct costs of animal disease, including animal mortality, morbidity, and associated response costs. However, such approaches often fail to capture the wider, dynamic market impacts that could arise. The duration of these market dislocations could last well after an initial disease outbreak. More generally, current approaches also muddle definitions of indirect costs, confusing debate on the scope of the totalities of disease-induced economic impacts. The aim of this work was to clarify definitions of indirect costs in the context of animal diseases and to apply this definition to a time series methodological framework to estimate the indirect costs of animal disease control strategies, using a foot and mouth disease (FMD) outbreak in Scotland as a case study. Time series analysis is an econometric method for analyzing statistical relationships between data series over time, thus allowing insights into how market dynamics may change following a disease outbreak. First an epidemiological model simulated FMD disease dynamics based on alternative control strategies. Output from the epidemiological model was used to quantify direct costs and applied in a multivariate vector error correction model to quantify the indirect costs of alternative vaccine stock strategies as a result of FMD. Indirect costs were defined as the economic losses incurred in markets after disease freedom is declared. As such, our definition of indirect costs captures the knock-on price and quantity effects in six agricultural markets after a disease outbreak. Our results suggest that controlling a FMD epidemic with vaccination is less costly in direct and indirect costs relative to a no vaccination (i.e., “cull only”) strategy, when considering large FMD outbreaks in Scotland. Our research clarifies and provides a framework for estimating indirect costs, which is applicable to both exotic and endemic diseases. Standard accounting CBAs only capture activities in isolation, ignore linkages across sectors, and do not consider price effects. However, our framework not only delineates when indirect costs start, but also captures the wider knock-on price effects between sectors, which are often omitted from CBAs but are necessary to support decision-making in animal disease prevention and control strategies.

## Introduction

Animal diseases represent threats to the environment, animal welfare, public health, and the economy. Livestock diseases contribute to losses via increased mortality, reduced productivity, control costs, loss in trade, decreased market value, and food insecurity (1). The economic and social impacts of livestock disease have been recognized globally, in both developed and developing countries (2). Quantifying the economic impact of an animal disease outbreak is important in support of prevention and control decisions for improved animal health.

The economic costs of animal disease can be categorized as either direct or indirect losses.

Over the last decade, the direct cost of zoonotic diseases has been estimated at more than $20 billion and indirect losses at over $200 billion to affected economies as a whole (3). This highlights that indirect costs are an important aspect of the economic impact of an animal disease outbreak, and as these estimates suggest, can be larger in magnitude than direct costs (3–5). While direct disease costs are important, indirect costs are also of concern (6) because the costs of disease do not stop at the farm-gate, within the agricultural sector, or after disease-freedom is declared. Disease can affect a wide range of sectors of the economy including rural business and tourism (7). However, few studies evaluate the full economic cost of disease outbreaks (8). Often only farm costs are considered and indirect impacts are not included (9, 10). There is a danger that estimates of economic costs of animal disease fail to capture indirect costs and may underestimate the true costs of an outbreak. It is important to understand the full economic cost of animal disease outbreaks, and to achieve this, economic disease cost frameworks must include indirect costs. This is essential to support holistic decision-making of disease prevention and control strategies because producers and policymakers need to be aware of the broader disease impacts to improve animal health welfare strategies and policy. This will be particularly important if alternative policy options lead to significantly different indirect cost outcomes and hence different decision choices than would otherwise be indicated.

At the same time, even where indirect costs are considered in the analysis, the definitions of direct and indirect costs of animal disease outbreaks vary in the literature (as described in Table 1). Some studies do not categorize economic costs as either direct or indirect, while others do not explicitly define direct and indirect costs (17). This non-exhaustive summary table highlights the inconsistency in the definition of direct and indirect costs making it difficult to quantify and compare the economic impact of livestock diseases. In particular, prevention and control costs are allocated as either direct or indirect costs, depending on the individual interpretation. The distinction between direct and indirect economic losses of animal diseases is unclear and subjective. Often there are a lack of data and an analytical framework to capture indirect costs. Hence, there is a need for a more systematic and unified framework on which to estimate and assess the economic impact of animal diseases (18). It is important to categorize direct and indirect costs more objectively to help determine who the economic impact of alternative animal disease scenarios likely fall upon.

**Table 1:** Non-exhaustive literature review summary of the definitions of direct and indirect components of animal disease costs.

**Direct costs**	**Indirect costs**	**Source**
Visible production losses (e.g., death, lower yield, and reduced growth) and invisible losses (e.g., reduced fertility and changes to herd structure) losses	Disease control costs Revenue foregone from restricted market access	(11)
Disease control costs	Export losses	(12)
Disease detection, confirmation, and control costs	Revenue foregone from trade restrictions Production losses beyond the agricultural sector Farmer losses taking into account market value and compensation received	(13)
Loss in profitability	Disease control costs	(14)
Disease losses that are experienced at the herd level on farm	Public expenditures and losses that occur beyond the farmgate	(9)
Disease control and prevention costs	Export losses	(4)
Losses to agriculture, the food industry, the public sector, and consumers	Losses to other sectors in the supply chain and tourism	(15)
Disease management and carcass disposal costs	Net economic welfare of the disease to producers, processors, and consumers.	(16)

A country's animal disease status changes over time. For this reason, we assume direct costs are the sum of losses from the first confirmation of a notifiable disease outbreak until disease freedom is declared (19). Accordingly, indirect costs are defined as the economic loss incurred in affected commodity markets (e.g., domestic and international trade) and in other sectors (e.g., tourism) *after* disease freedom is declared. Applying this definition, indirect costs are related to knock-on effects (i.e., shocks) in markets as a result of changes in prices and quantities for producers and/or consumers, which can also be described as revenue foregone, after disease freedom. Using disease status as a marker, our definition of indirect costs objectively differentiates when direct costs end and indirect costs begin to avoid double counting.

A range of models are available for assessing the economic cost of livestock diseases (20). Based on our definition, indirect effects capture the substitution and displacement in markets as a result of changes to price and output in agriculture and tourism sectors (7). Capturing such dynamics is challenging and there is a need for models that encapsulate the impacts of a disease outbreak in multiple agricultural markets and linkages with non-agricultural sectors (21). Traditional cost benefit analyses (CBAs) based on farm accounts and partial budgets cannot capture such dynamics, and as such partial equilibrium (PE) (4, 22–24) and computable general equilibrium (CGE) (7, 25, 26) models are being used to estimate the indirect knock-on effects of animal disease. A PE model is based on supply and demand relationships to evaluate the impact of a shock, such as a disease outbreak, on one sector of the economy assuming the rest of the economy is fixed. This approach is thus simplified and ignores any sector interactions. On the other hand, CGE models simulate how a multi-sector economy might respond to a shock until equilibrium is restored, with linkages between different sectors. While CGE models link multiple sectors and can represent the entire economy they also rely on economic data and in some cases estimates of elasticities^1^ to parameterize market responses. Hence, a weakness of these models is a reliance on estimates of elasticities which are often outdated or “guesstimated,” if available at all, which might affect the model's performance and estimation. Therefore, in the absence of good data or if elasticities cannot be estimated, demand and supply relationships are based on assumptions.

An alternative and complementary approach to PE and CGE modeling is times series analysis. Time series modeling (27) is an econometric method for identifying patterns and representing statistical relationships between data series ordered over time, and forecasting to predict using observed data. Time series models have been cited extensively in the literature including in the disciplines of economics, mathematics, epidemiology, finance, meteorology, engineering, and natural sciences to name just a few. However, their application in estimating the cost of animal disease is somewhat limited (28, 29). In these examples, time series models have estimated the impact that a disease outbreak is likely to have on markets. The type of time series model selected depends on the underlying statistical properties of the data (30). Time series models assume data are stationary, such that the mean, variance, and autocorrelation structure do not change over time. Stationary properties of the data define which time series model to use. Vector autoregressive (VAR) models (31) are a multivariate generalization of a univariate autoregressive model, in which each variable is a linear function of past values of itself and other variables. Alternatively, autoregressive distributed lag models (32) are based on regression equations to predict using current and past values of time series. When cointegration is detected, i.e., long-run relationship between variables, a vector error correction model (VECM) is the most appropriate model to represent the data. A VECM estimates long-run equilibrium relationships and short-run dynamics between data series over time (33). Impulse response functions (IRFs) (34) are a useful tool for forecasting and determining the relationship between variables over time until a shock dissipates. An IRF describes the change in a variable over a time after a shock in another variable. IRFs lend themselves to modeling a disruption to supply chain shock, i.e., animals culled following an outbreak, and simulating the response of such a shock in other variables. Once obtained, IRF coefficients can be interpreted as elasticities (35) on which to estimate price and quantity changes for estimating indirect costs, i.e., the economic losses incurred in markets after disease freedom is declared.

The culling of animals for disease control reduces their supply, disrupting domestic meat production. Economic theory assumes that the slaughter of animals will lead to a supply shortage affecting producers and consumers by increasing the prices consumers pay for commodities (36). During an exotic disease outbreak, an export ban would be triggered which is likely to put downward pressure on prices as meat destined for export would remain within domestic markets. While this might be the case during an outbreak, what will happen in markets after disease freedom is declared and how much prices and quantities will adjust by? Time series analysis can help with this, using market data, to estimate such price and quantity changes, i.e., market response, without relying on estimates of elasticities from the literature. Hence, a more data-focused time series model can capture the relationship between prices and substitution effects between markets to compliment and feed into more comprehensive yet computationally demanding assumption-based models, such as CGE models, which rely on good data from existing literature.

The overall aim of this work was to outline the steps necessary to estimate indirect costs, i.e., the economic losses incurred in markets after disease freedom is declared using a time series model. We apply this in the context of a modeled Foot and Mouth Disease (FMD) in Scotland. FMD is considered one of the most economically significant livestock diseases globally due to its impact on production, as a barrier to international trade and high control/stamping out costs (8). While the direct costs of FMD in Scotland have been estimated, there is a need for indirect costs to also be estimated (19). Therefore, our paper seeks to remedy this by estimating the indirect costs of a hypothetical FMD outbreak in six of Scotland's important agricultural commodity markets, (i.e., beef, pork, lamb, chicken, milk, and feed wheat), and, by this provide a more objective definition of indirect costs using a time series modeling framework. We considered agricultural commodity markets that were thought to be most affected by an FMD outbreak. The indirect economic impacts are likely to be felt much more widely than this study attempts to quantify.

While FMD is likely to affect international trade and tourism, the data to support such analysis are not available at an appropriate resolution. Hence, our paper focusses on the domestic supply side evaluating indirect costs incurred by producers after a disease outbreak is over as an illustration of the method. The distribution of indirect costs was compared to direct costs on alternative FMD control strategies in Scotland. We assess the potential impact of vaccine stock scenarios on indirect costs on decision outcomes in a future outbreak and so the suitability of time series for contribution to decision support. Vaccine capacity is important (37, 38) and vaccination plays a key role in large outbreaks of FMD in terms of the epidemiological benefit (39) and direct economic costs (19). Hence, this paper evaluates the indirect costs of alternative levels of vaccines stocks to compliment previous work (19). Our indirect cost estimation framework can be applied to other animal disease outbreaks in Scotland, the UK or elsewhere. The paper provides insights into an econometric method which quantifies broader knock-on effects of notifiable animal disease that affect production and trade after an outbreak is over which are often overlooked.

## Materials and Methods

**Figure 1:**
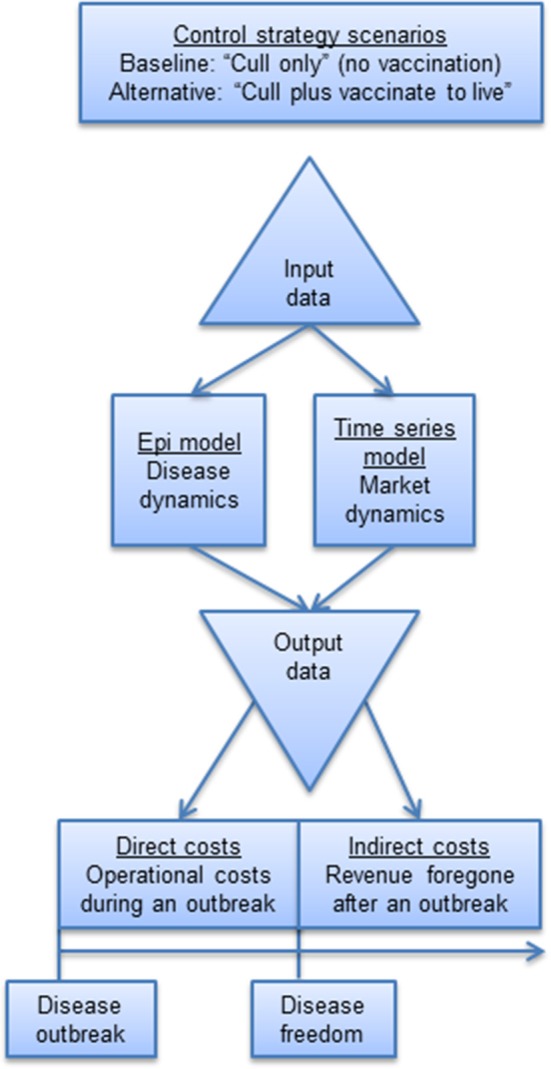
Economic cost modeling framework.

Our indirect cost methodology was demonstrated in the context of a FMD outbreak in Scotland. The indirect cost modeling framework (Figure 1) for estimating indirect costs involved the following four steps: (i) collection of input data; (ii) selection and specification of a time series model to simulate market dynamics; (iii) simulation of disease dynamics by way of an epidemiological model; and (iv) estimation of indirect costs based on integrating output from the time series model and epidemiological model under alternative disease control strategies. This methodological framework is described below.

### Overview of Foot and Mouth Disease

FMD is a highly contagious viral disease affecting ruminants, including cows, sheep and pigs. Globally, FMD is estimated to cost endemic countries between $6.5 and $21 billion annually, due to visible production losses and vaccination costs in endemic countries (8). In addition, previously disease-free countries incurred outbreak costs of between $0.5 billion and $10 billion following an outbreak, i.e., between 0.2 and 0.6% of GDP (8). These losses make FMD one of the most economically important livestock diseases. In the UK, FMD is a notifiable exotic disease, with the last outbreak in 2007 estimated to have cost the British livestock sector over £100 million and the government £47 million (40). However, a larger, costlier outbreak occurred in 2001 generating losses of over £8 billion (41). During the 2001 outbreak, the first case of FMD was confirmed on 20 February and the disease was eradicated by the end of September 2001, by which time more than 6 million animals were slaughtered (41).

Animal health and welfare is a devolved issue in the UK, meaning the Scottish Parliament and Scottish Government have responsibility for the health and welfare of animals in Scotland. FMD is a notifiable disease and control mechanisms include movement bans and restrictions of the marketing of milk and meat products during an outbreak. The principal control method to eradicate FMD, as required under EU and national law, is the slaughter of affected animals (i.e., infected animals and dangerous contacts) to prevent any further spread of the virus. Vaccination is also an important tool in controlling FMD during an outbreak. However, preventative vaccination is banned under EU law, but the Scottish Government considers emergency vaccination a disease control strategy during an outbreak (42). The presence of a notifiable exotic disease, such as FMD, will result in the UK losing its FMD disease freedom status and trigger an export ban until disease freedom is declared. The loss of export trade may persist beyond disease freedom should importing countries adopt a precautionary approach.

### Data Collection

Monthly agriculture commodity price and quantity data between January 2004 and December 2016 (*n* = 156 observations) were gathered from various sources for this study (see Table 2). Producer prices were adjusted for inflation using the producer price index (47) to reflect real prices in 2011, the year in which the modeled hypothetical FMD outbreak occurred. Some data series were only available at either the UK or Great Britain level, consequently these data were adjusted to reflect Scottish prices or Scotland's share of the UK's or Great Britain's volume of production. The prices of UK pork, lamb, chicken, milk, and feed wheat were adjusted by 0.99 to reflect prices in Scotland relative to UK levels (48). Wholesale milk production was adjusted to reflect Scotland's share of the UK's milk production (49). Scotland's production of feed wheat was also adjusted to reflect Scotland's share of Great Britain's production (50). Scottish cattle, pig and lamb slaughtered in Scotland was adjusted to reflect Scottish livestock slaughtered in the rest of the UK (i.e., beef: 5%, pig; 55%, lamb: 15%) (51).

**Table 2:** Description of monthly price and quantity data series.

**Data series** **(*Acronym*)**	**Description of data**	**Units**	**References**
Price of beef (*PBeef*)	Average monthly farmgate price of Scottish steers (deadweight price)	£ Per ton	(QMS 2017, personal communication, 20 October)
Price of pork (*PPork*)	Average monthly farmgate price of pork in the UK (deadweight price)	£ Per ton	(QMS 2017, personal communication, 20 October)
Price of lamb (*PLamb*)	Average monthly farmgate price of lamb in the UK (deadweight price)	£ Per ton	(QMS 2017, personal communication, 20 October)
Price of chicken (*PChicken*)	Average monthly wholesale price of chicken in the UK (roasters 2050g and under 2450g)	£ Per ton	(Defra 2017, personal communication, 1 September)
Price of milk (*PMilk*)	Average monthly farmgate price of milk in UK	£ Per liter	(43)
Price of feed wheat (*PWheat*)	Average monthly farmgate price of feed wheat in the UK	£ Per ton	(44)
Quantity of cattle (*QCattle*)	Quantity of cattle (including finished and culled cattle) of Scottish origin slaughtered (carcase weight)	Ton	(Scottish Government 2017, personal communication, 3 October)
Quantity of pig (*QPig*)	Quantity of pigs (including sows and boars) of Scottish origin slaughtered (carcase weight)	Ton	(Scottish Government 2017, personal communication, 3 October)
Quantity of sheep (*QSheep*)	Quantity of sheep (including lambs and ewes) of Scottish origin slaughtered (carcase weight)	Ton	(Scottish Government 2017, personal communication, 3 October)
Quantity of chicken (*QChicken*)	Quantity of poultry of Scottish origin slaughtered (carcase weight)	Ton	(Scottish Government 2017, personal communication, 3 October)
Quantity of milk (*QMilk*)	Quantity of wholesale milk produced in the UK	Liters	(45)
Quantity of feed wheat (*QWheat*)	Quantity of Scottish feed wheat (animal feeding stuff) production in Great Britain	Ton	(46)

Scotland was assumed to be a closed economy in terms of economic impacts on domestic supply because trade (i.e., exports and import) data were not available at an appropriate monthly resolution to determine the indirect cost after disease freedom is declared. Consumer demand was assumed not to be affected because FMD is not a zoonosis and there was not sufficient model power to include retail market data series.

### Time Series Model Selection

**Figure 2:**
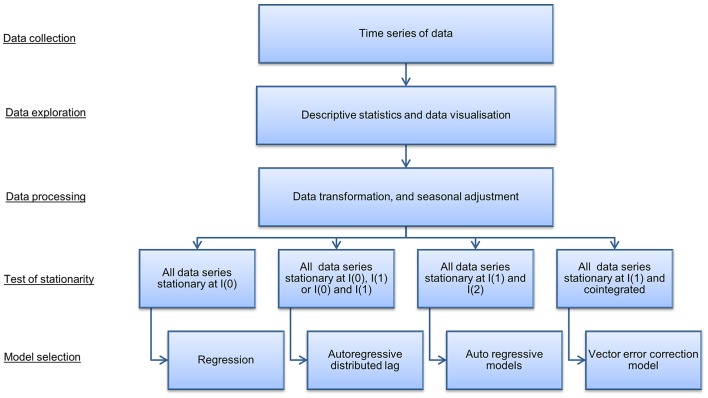
Summary of steps in time series model selection. Source: Adapted from Wooldridge (52) and Johnston and DiNardo (53).

A time series model was used to quantify the indirect costs, i.e., the economic losses incurred in markets after disease freedom is declared, in domestic commodity markets associated with an FMD outbreak in Scotland. The steps for selecting the most appropriate time series model are presented in Figure 2 [Adapted from Wooldridge (52), Johnston and DiNardo (53)]. Following data gathering and transformation, the order to which data series are integrated and the presence of cointegration determines the times series model selected. In our case, a VECM was selected and an IRF evaluated market dynamics resulting from a hypothetical outbreak for the estimation of indirect costs.

### Data Exploration and Processing

Descriptive statistics and plotting were used to summarize and visualize the characteristics and patterns in the data series, including the presence of seasonal variation. Seasonal adjustments were performed by estimating and removing seasonality from the data to understand underlying trends and movement in the data over time, masked by seasonal variation (54–57). Data were expressed in natural logarithms to ensure the series were on a consistent scale.

Following the methodological framework (Figure 1), data series were decomposed into seasonal, trend, and residual components (Figure S1). The seasonally decomposed data series suggest that the pattern of seasonality is similar across months for each variable. Therefore, seasonality was removed additively before modeling the data. Following removal of seasonality, data were expressed in natural logs to ensure the series were on a consistent scale.

### Testing of Stationarity and Cointegration

Stationarity is an underlying statistical property of data required for time series analysis. A stationary process is such that the mean, variation, and autocorrelation in the structure of the data do not change over time. A trend in the mean due to the presence of a unit root or deterministic trends are causes that violate the underlying assumption of stationarity.

The data series were tested for stationarity (i.e., the presence of unit roots) using the augmented Dickey-Fuller [ADF; (58)] test to detect the order of integration, a metric describing a unit root process in time series analysis. A time series, Y_*t*_, is integrated of order 0, or at the level, if Y_*t*_ ~ *I*(0) is stationary. *Y*_*t*_ is integrated of order 1, denoted by *I*[1), if it is not stationary but the first difference (i.e., *Y*_*t*_ − *Y*_*t*−1_) of the series is stationary. If *Y*_*t*_ is non-stationary, but *Y*_*t*_ ~ *I*(d) such that *d* > 0 is stationary, then the data series is integrated of order *d*. Agricultural commodity market data are assumed to be stationary but typically such data exhibit non-stationary behavior (59).

Examining the stationary process of the data and cointegration between the time series will determine with which model to analyse the data [Figure 2: Adapted from Wooldridge (52) and Johnston and DiNardo (53)]. Cointegration is a statistical property that identifies long-run relationships between data series. Data series are cointegrated if all the series are integrated of order 1, (i.e., *I*(1)) and a linear combination of these series are integrated of order 0 (i.e., *I*(0)). If cointegration is present, this suggests there is an equilibrium relationship in the long-run, although the series may diverge from that equilibrium in the short-run. Johansen's trace test (33) is a statistical procedure to determine whether two or more *I*(1) time series are cointegrated. The Johannsen's trace test was used because it is robust to skewness and excess kurtosis. In the case that cointegration is present a vector error correction model is selected, which models both short and long-run relationships jointly in multiple data series. In cases where no cointegration is detected alternative models, such as autoregressive and autoregressive distributed lag models, are appropriate [Figure 2: Adapted from Wooldridge (52) and Johnston and DiNardo (53)].

### Model Selection

Statistical testing for stationarity and cointegration described above and outlined in Figure 2 [Adapted from Wooldridge (52) and Johnston and DiNardo (53)] identified that a VECM model was appropriate. The Results section describes the outcome of the statistical testing. A VAR model of order *p* (where *p* is the number of lags), or a VAR(*p*), combined with an error correction model can be modeled as a VECM with *p* − 1 lags, i.e., VECM(*p* − 1) (33). According to the Granger theorem (60–62), a general multivariate VECM(*p* − 1) with K endogenous variables, an intercept, *u*, and time trend, δ*t*, takes the form:

(1) $$\begin{array}{l} \Delta Y_{t}=\mu +\delta t+\sum_{l=1}^{p-1}\Gamma_{l}\Delta Y_{t-l}+{\prod Y}_{t-1}{+\;\varepsilon }_{t} \end{array}$$

where, *Y*_*t*_ is a *K* × 1 vector of *K I*(1) endogenous variables such that the first difference is *Y*_*t*_ = *Y*_*t*_ − *Y*_*t*−1_. The number of lags is denoted by *l* (where, *l* = 1, …, *p* − 1) and *t* is the time period. *u* is a *K* × 1 parameter vector associated with the intercept and δ is a *K* × 1 parameter vector associated with a time trend, *t*. The deterministic regressors, *u* and δ contribute to both the short and long-run components of *Y*_*t*_. Γ_*l*_ is a *K* × *K* matrix of short-run dynamic adjustment coefficients at lag *p* − 1 of *Y*_*t* − *l*_. ∏ is a *K* × *K* error correction matrix and the long-run equilibrium relationship among *Y*_*t*_ is determined by the rank, *r*. The matrix ∏ contains long-run relationships assuming there is a reduced rank of 0 ≤ *r* ≤ *K* it follows that ∏ = −αβ′. The strength of cointegrating relationships is determined by α and β′. Where, α is a *K* × *r* matrix of speed of adjustment to equilibrium coefficients of which *K* variables adjust to error correction terms at varying speeds and β′ is a *r* × *K* matrix of long-run cointegration coefficients. ε_*t*_ is a *K* × 1 vector of independently and identically distributed errors over time with a mean of 0 and covariance matrix, Σ_ε_. Following this, a VECM(*p* − 1) in (1) can be written as:

(2) $$\begin{array}{l} \Delta Y_{t}=\mu +\delta t+\Gamma_{1}\Delta Y_{t-1}+\Gamma_{2}\Delta Y_{t-2}+\ldots \\ \;+{\Gamma_{p-1}\Delta Y_{t-\left(p-1\right)}+\alpha \beta^{{}^{\prime }}Y}_{t-1}{+\;\varepsilon }_{t} \end{array}$$

where, $\Gamma_{j}=-\overset{p}{\underset{j=l+1}{\sum }}\prod_{j}$. Based on the VECM(*p* − 1) (2), an IRF was estimated to evaluate how 12 endogenous variables (i.e., *PBeef*, *PPork, PLamb, PChicken, PMilk, PWheat, QCattle, QPig, QSheep, QChicken*, Q*Milk*, and *QWheat*) responded to 3 impulses, or shocks, (i.e., *QCattl*e, *QPig* and *QSheep*) at a particular point in time and subsequent periods. An IRF gives the response of the *k*th variable when a system is shocked by one standard-deviation in the *j*th variable, and the matrix ψ allows for alternative responses in different variables. When data are expressed in natural logarithms, the IRF coefficients represent the percentage change in the *k*th variable when the system is shocked by a 1% change in the *j*th variable. As such, the values of IRF coefficients are interpreted as elasticities, namely IRF elasticities. The IRF estimates the response of endogenous variable *k*, *Y*_*k, t*+*n*_, to a one-time impulse, or shock, in variable *j*, *Y*_*j, t*_, from time *t* to time *t* + *n*. Where, n = 0,…, N is the number of time periods specified over which the endogenous response variable evolves with all other endogenous variables at time *t* or earlier held constant. The IRF is expressed as:

(3) $$\begin{array}{l} Y_{k,\;t+n}=\sum_{i=0}^{\infty }\psi_{i}\varepsilon_{t+n-i} \end{array}$$

(4) $$\begin{array}{l} {\left\{\psi_{n}\right\}}_{k,\;j}=\frac{\delta Y_{k,t+n}}{\delta \varepsilon_{j,t}} \end{array}$$

where, *Y*_*k, t*+*n*_ is a function of current and lagged impulses, or shocks, and accordingly an IRF represents the adjustment process and impacts of a shock over time. The coefficients in ψ_*kj*_ are the impulse response functions, where ψ is a *n x k* matrix of *j* matrices depending on the number of response variables, *Y*_*k, t*_, impulses, *Y*_*j, t*_, and n, the number of specified time periods over which the response variables evolve and time periods after which the impulse dissipates, *i*. A generalized IRF assumes that a shock occurs at a single point in time such that shocks in different variables are independent and invariant to the ordering of variables (34). If correlation between the error terms is detected it suggests that a shock in one variable is likely to be accompanied by a shock in another variable and an orthogonalized IRF is used to model structural shocks.

### Epidemiological Model

We used the Warwick FMD model to simulate the spread of FMD following a hypothetical introduction in Scotland in June 2011 and simulate various scenarios of vaccination (19). This model is a fully stochastic, spatial, farm-based model that was developed and used during the FMD epidemic in 2001 in Great Britain (63–67) and was later modified to represent the Scottish livestock industry (39). Although transmission of FMD is restricted to all farms with cattle, sheep or both, disease control activities implemented in the model will involve farms showing at least one animal susceptible (including pigs and deer). The model further assumes FMD individuals will pass through four epidemiological states: susceptible; infected, but not infectious; infectious; or reported infected and thereby culled. Following the introduction of the virus in a given *j*th premises, the model assumes that each *i*th premises is infected with a daily probability *M*_*i*_ depending on its own susceptibility *S*_*i*_ and on the transmissibility *T*_*j*_ of the surrounding *j* premises such that:

$$\begin{array}{l} M_{i}=1-\exp\left(-S_{i}\underset{i\neq j}{\sum }T_{j}K\left(d_{ij}\right)\right) \end{array}$$

where *S*_*i*_ and *T*_*i*_ depend on the species (i.e., cattle and sheep) and on the related herd size on premises (63–67). The component *K*(*d*_*ij*_) is the so-called “transmission kernel function” and determines the scaling factor on the rate at which infected premises may infect susceptible ones as a function of inter-farm distance *d*_*ij*_.

A baseline scenario of a “cull only” (i.e., no vaccination) vs. alternative scenarios of “cull plus vaccinate to live” policy was simulated. The availability of vaccine stocks at the start of an outbreak was considered assuming only cattle were vaccinated and that vaccinated animals would become immune to infection after 4 days (42). As in previous work (19, 39), we made the conservative assumption that during this 4-day delay, all cattle are completely susceptible and if infected, the disease progresses in the same way as for non-vaccinated cattle. We also considered that not all cattle present on vaccinated farms would become totally immune, with 10% of the cattle remaining totally susceptible to infection and able to transmit the virus to farms that were not vaccinated (65). In line with current regulations in place in Scotland, we assumed that the vaccination campaign would start 14 days after the disease is first detected, allowing the decision to vaccinate to be taken, the doses of vaccine to be received from the appropriate vaccine bank and vaccination teams to be mobilized and actively deployed in the field. Once the decision to vaccinate has been made, vaccination would be implemented within a 10-km-radius buffer around each IP and carried out within the recommended 24 h (42).

The model simulated the effects of the Scottish Government's FMD contingency plan under alternative vaccine stock scenarios (i.e., initial vaccine stocks ranged from 100,000 to 5 million doses as in Porphyre et al. (19). Briefly, we considered that, for each vaccine stock scenario, 10,000 epidemics were simulated assuming that FMD is introduced in a single susceptible herd and spread silently to four additional herds due to delays in detecting new incursion events. Although we arbitrarily considered that outbreaks will be initiated with five infectious premises, this was based on the fact that: (1) it is unlikely for cattle farms to remain undetected for long period of time given the high awareness of farmers to the disease in the UK due to the traumatic experience during the 2001 outbreak; (2) the noticeable symptoms of FMD infection in cattle (68) and; (3) the implementation of the standstill regulations which would limit the spread of FMD due to animal movement (69). As such, the spread of FMD is likely to be mostly driven by local spread and affect a relatively small number of farms within a short period. Over all simulations, we used the same set of all initially infected herds. These were located in the county of Ayrshire, which has a high density of premises and animals, and has been previously identified as an area where there is potential for extensive initial spread (39), and hence represent the worst case scenario for FMD spread in Scotland.

Output data from the epidemiological model included the number of animals (i.e., cattle, pigs, and sheep) culled for disease control purposes, which informed the estimation of indirect costs. Direct economic costs, i.e., the economic losses incurred in markets before disease freedom is declared, were estimated from the epidemiological model data and are published (19).

### Indirect Cost Estimation

The indirect costs, i.e., the economic losses incurred in markets after disease freedom is declared, associated with a FMD outbreak in Scotland were estimated by integrating output from the time series model (i.e., IRF elasticities) and epidemiological model (i.e., number of animals culled inputs into the indirect costs time series model).

The IRF elasticities capture the changes in the levels of prices and quantities in six commodity markets (i.e., beef, pork, lamb, chicken, milk, and feed wheat) following the culling of 1% of animals (i.e., cattle, pig, and sheep). The IRF elasticities capture the adjustment of prices and quantities to a long-run equilibrium until the effect of the shock dissipates over time. We identified the period in which the impact of the shock dissipated, i.e., the change in the IRF elasticities tended to zero. This determines up to what period to sum the IRF elasticities to quantify the total economic impact of the animals culled.

To estimate the total impact of the supply shock, the IRF elasticities were multiplied by the epidemiological shock (i.e., number of animals culled as output from the epidemiological model) as a proportion of the national production herd [1,803,937 cattle; 389,995 pigs; and 6,801,134 sheep in June 2011; (70)]. As a result, the IRF elasticities reflect the total economic impact of a supply shock taking into account the size of the outbreak in terms of animals culled.

The indirect costs, *IC*_*s*_, of alternative vaccination strategies, *S*, and six domestic commodity markets (i.e., beef, pork, lamb, chicken, milk, and feed wheat), *i*, were estimated. The indirect costs are associated with price and quantity changes, i.e., change in revenue or revenue foregone, in each market, *i*, as a result of a supply shock of animals culled, *j*, after disease freedom is declared:

(5) $$\begin{array}{l} IC_{s}=\;\sum_{j=1}^{3}\sum_{i=1}^{6}\left(\;P_{i,d}*Q_{i,d}\right)-\left(P_{i,t}*Q_{i,t}\right) \end{array}$$

where *i* denotes commodity markets for beef, pork, lamb, chicken, milk, and feed wheat, and *j* represents cattle, pig and sheep culled. *P*_*i*_ and *Q*_*i*_ are the price and quantity in the *i*th commodity market, respectively. *t* denotes the period before the outbreak and *d* is the period after disease freedom is declared until the supply shock dissipates. To quantify the total indirect costs across the six markets, the change in revenue is summed across the commodity markets for animals culled for alternative scenarios, *S*.

(6) $$\begin{array}{l} IC_{s}=\;\sum_{j=1}^{3}\sum_{i=1}^{6}\left(P_{i,t}*\left(1+\left(El_{Pi,j}\right)\right)\right)*\left(Q_{i,t}*\left(1+\left(El_{Qi,j}\right)\right)\right)-(P_{i,t}*Q_{i,t}) \end{array}$$

where *El*_*Pi, j*_ is the IRF elasticity of price of the *i*th market, *P*_*i*_, with respect to the *j*th species culled, and the *El*_*Qi, j*_ is the IRF elasticity of quantity of the *i*th market, *Q*_*i*_, with respect to *j*th species culled. The IRF elasticities, estimated from the time series model, capture proportional changes in the levels of price and quantity changes as a result of animals culled. Finally, the total economic cost is the sum of indirect and direct costs.

## Results

The objective of this paper was to demonstrate a method for estimating the indirect costs, i.e., the economic losses incurred in markets after disease freedom is declared, under alternative disease control strategies using time series analysis.

### Data Exploration and Processing

**Figure 3:**
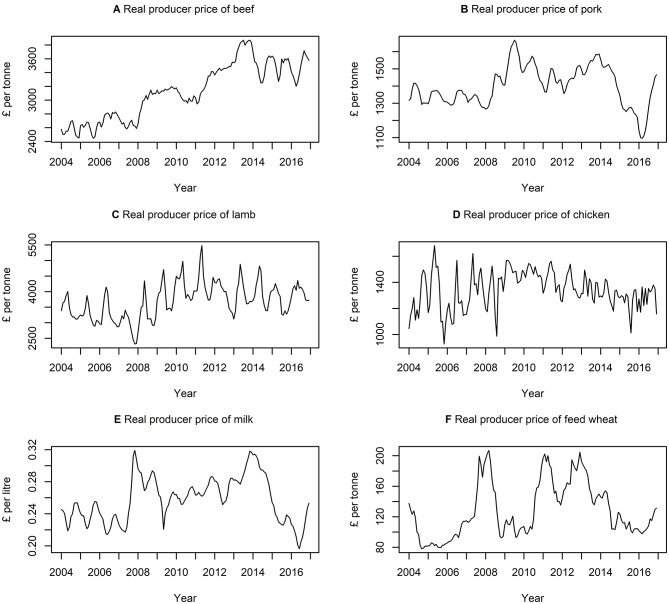
Times series of real producer prices of **(A)** beef, **(B)** pork, **(C)** lamb, **(D)** chicken, **(E)** milk, and **(F)** feed wheat between January 2004 and December 2016, inclusively.

**Figure 4:**
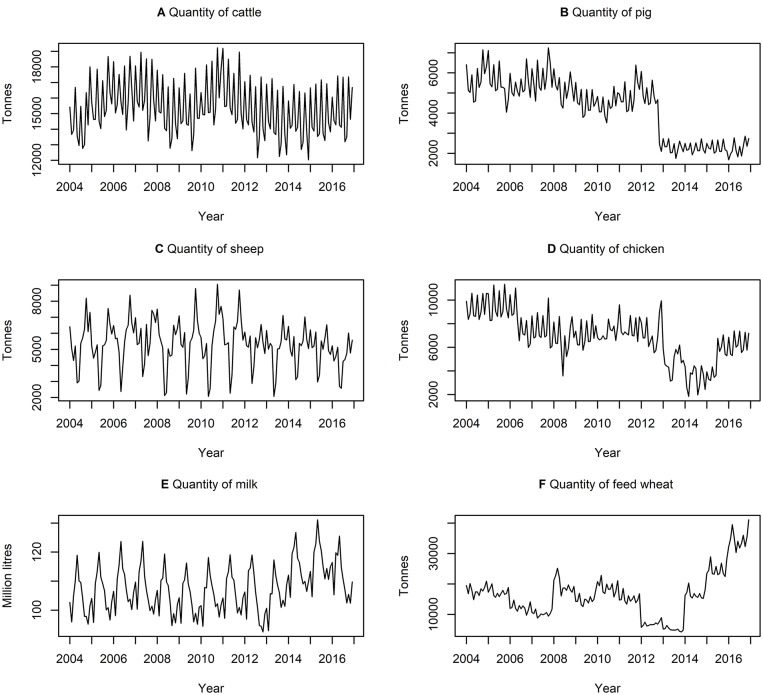
Time series of quantities of **(A)** cattle **(B)** pig **(C)** sheep **(D)** chicken **(E)** milk produced and **(F)** feed wheat produced between January 2004 and December 2016, inclusively.

Table 3 presents descriptive statistics for the data series. The lowest and highest variation, according to the coefficient of variation (i.e., ratio of standard deviation to the mean) is quantity of milk produced and quantity of feed wheat produced, respectively. On average, there is a higher variation in the quantity of commodities rather than the price of commodities. Figures 3, 4 show the data series of prices and quantities, respectively, plotted over time.

**Table 3:** Summary statistics of monthly agriculture commodity price and quantity data between January 2014 and December 2016 (*n* = 156 observations).

**Data series** **(units)**	**Minimum**	**Maximum**	**Median**	**Mean**	**Standard deviation**	**Coefficient of variation**
Price of beef (£ per ton)	2,446	3,870	3,132	3,126	400.32	0.128
Price of pork (£ per ton)	1,096	1,667	1,401	1,400	114.34	0.082
Price of lamb (£ per ton)	2,317	5,476	3,711	3,694	566.18	0.153
Price of chicken (£ per ton)	928	1,681	1,347	1,341	140.27	0.105
Price of milk (£ per liter)	0.20	0.32	0.26	0.26	0.03	0.109
Price of feed wheat (£ per ton)	78	207	117	129	36.18	0.281
Quantity of cattle (ton)	12,034	19,226	14,996	15,353	1,746.00	0.114
Quantity of pig (ton)	1,679	7,246	4,580	4,230	1,502.50	0.355
Quantity of sheep (ton)	2,073	9,048	5,376	5,319	1,429.50	0.269
Quantity of chicken (ton)	1,841	11,337	6,868	6,892	2,017.50	0.293
Quantity of milk (ton)	92,533,221	131,099,341	106,746,572	107,461,126	7,978,941.54	0.074
Quantity of feedwheat (ton)	4,282	41,042	16,139	16,510	7,560.56	0.458

### Test of Stationarity and Cointegration

The ADF unit root test (58, 71) was conducted on each data series to determine to what degree data series are integrated. The ADF test indicated that 11 of the 12 data series contained were stationary at *I*(1)), except *QSheep* (i.e., quantity of sheep) which is stationary at the level, i.e., *I*(0), at the 5% level of significance. While a VECM requires all variables to be stationary at *I*(1), in systems with three or more series a VECM is appropriate providing at least two of the variables are stationary at *I*(1) (72). Therefore, *QSheep* does not impact the validity of our VECM.

The next step was to test for cointegrating relationships between the variables. The Trace statistics (Table 4) tests the null hypothesis that there are no cointegrating relationships (i.e., *r* = 0) against the alternative that there is at least one cointegrating relationship (i.e., *r* ≥ 1).

**Table 4:** Johansen cointegration trace test for determining the number of cointegrating relationships, r.

**Null hypothesis**	**Alternative hypothesis**	**Trace statistic**	***p-*value**
*r* = 0	*r* ≥ 1	373.15	<0.001
*r* ≤ 1	*r* ≥ 2	292.26	0.02
*r* ≤ 2	*r* ≥ 3	237.76	0.06
*r* ≤ 3	*r* ≥ 4	185.55	0.16
*r* ≤ 4	*r* ≥ 5	141.05	0.32
*r* ≤ 5	*r* ≥ 6	103.10	0.51
*r* ≤ 6	*r* ≥ 7	75.88	0.51
*r* ≤ 7	*r* ≥ 8	54.05	0.46
*r* ≤ 8	*r* ≥ 9	32.71	0.58
*r* ≤ 9	*r* ≥ 10	18.65	0.53
*r* ≤ 10	*r* ≥ 11	8.78	0.39
*r* ≤ 11	*r* = 12	3.20	0.07

Maximum-likelihood test of the cointegrating rank.

Trend assumption: Constant.

*Lag selection (lag = 1) based on Akaike information criterion (AIC) and Bayesian information criterion (BIC)*.

The trace statistic (Table 4) indicates at least two cointegrating relationships among our data series at the 5% level of significance. Since cointegration is detected, it was incorporated into our model because otherwise its omission contributes to misspecification error.

### Vector Error Correction Model

In this paper, a VECM is estimated because of the presence of stationarity in the data series at *I*(1) and cointegration. Long-run relationships were estimated using maximum likelihood for a VECM(1) with 12 endogenous data series (i.e., *K* = 12), one lag (i.e., *p* − 1 = 1), two cointegrating relationships (i.e., *r* = 2), and a constant deterministic regressor as shown in Allan et al. (7).

(7) $$\begin{array}{l} \left[\begin{array}{c} \begin{array}{c} \Delta In\;PBeef_{t}\\ \Delta In\;PPork_{t}\\ In\;PLamb_{t}\\ \Delta In\;PChicken_{t}\\ \Delta In\;PMilk_{t}\\ \Delta In\;PWheat_{t}\\ \Delta In\;QBeef_{t}\\ \Delta In\;QPork_{t}\\ \Delta In\;QSheep_{t}\\ \Delta In\;QChicken_{t}\\ \Delta In\;QMilk_{t} \end{array}\\ \Delta In\;QWheat_{t} \end{array}\right]=\left[\begin{array}{c} \mu_{1}\\ \vdots \\ \mu_{12\;} \end{array}\right]+\left[\begin{array}{ccc} \Gamma_{1\;1} & \cdots & \Gamma_{1\;12}\\ \vdots & \ddots & \vdots \\ \Gamma_{12\;1\;} & \cdots & \Gamma_{12\;12} \end{array}\right]\\ \left[\begin{array}{c} \begin{array}{c} \Delta In\;PBeef_{t-1}\\ \Delta In\;PPork_{t-1}\\ \Delta In\;PLamb_{t-1}\\ \Delta In\;PChicken_{t-1}\\ \Delta In\;PMilk_{t-1}\\ \Delta In\;PWheat_{t-1}\\ \Delta In\;QBeef_{t-1}\\ \Delta In\;QPork_{t-1}\\ \Delta In\;QSheep_{t-1}\\ \Delta In\;QChicken_{t-1}\\ \Delta In\;QMilk_{t-1} \end{array}\\ \Delta In\;QWheat_{t-1} \end{array}\right]+\left[\begin{array}{c} \alpha_{11}\\ \vdots \\ \alpha_{12\;1\;} \end{array}\begin{array}{c} \alpha_{12}\\ \vdots \\ \alpha_{12\;2\;} \end{array}\right]\;\left[\begin{array}{ccc} \beta_{1\;1} & \ldots & \beta_{1\;12}\\ \beta_{2\;1} & \ldots & \beta_{2\;12} \end{array}\right]\\ \left[\begin{array}{c} \begin{array}{c} In\;PBeef_{t-1}\\ In\;PPork_{t-1}\\ In\;PLamb_{t-1}\\ In\;PChicken_{t-1}\\ In\;PMilk_{t-1}\\ In\;PWheat_{t-1}\\ In\;QBeef_{t-1}\\ In\;QPork_{t-1}\\ In\;QSheep_{t-1}\\ In\;QChicken_{t-1}\\ In\;QMilk_{t-1} \end{array}\\ In\;QWheat_{t-1} \end{array}\right]+\left[\begin{array}{c} \varepsilon_{1\;t}\\ \vdots \\ \varepsilon_{12\;t} \end{array}\right] \end{array}$$

The estimated coefficient matrices for the VECM (7) are reported in Tables S1–S5.

### Impulse Response Function

**Figure 5:**
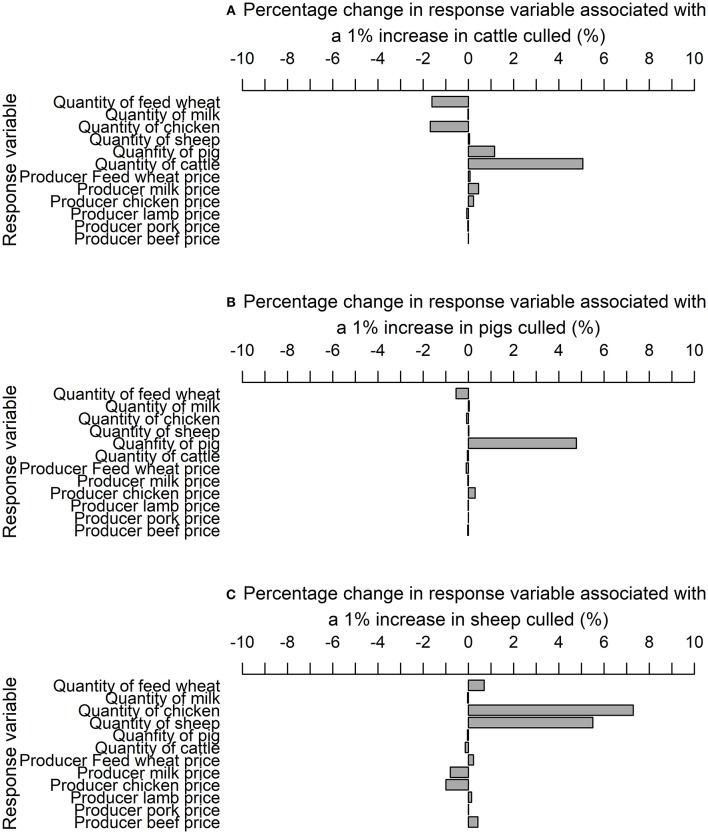
Impulse response elasticities associated with a 1% increase in the quantity of **(A)** cattle **(B)** pig and **(C)** sheep culled for disease control purposes.

Once a VECM was identified an impulse response function (IRF) was estimated to evaluate short-run dynamics. The correlation of the variance covariance matrix suggests there is little correlation between the coefficients (Table S6). In which case, a generalized IRF is the most appropriate IRF, which is invariant to the order of endogenous variables. A generalized IRF was estimated with 1,000 bootstrapped replications. The supply shocks dissipate in the market variables at different time horizons, from 2 to 13 months. The IRF elasticities capture the response of variables to a 1% shock decrease in the production of cattle (Figure 5A), pig (Figure 5B), and sheep (Figure 5C) due to animals culled until the supply shock dissipates. As described in the Materials and Methods, these IRF elasticities represent a 1% change because the data are expressed in natural logarithms. The IRF elasticities were multiplied by the hypothetical epidemiological shock as a proportion of the national production herd [1,803,937 cattle; 389,995 pigs; and 6,801,134 sheep in June 2011; (70)] to estimate the total impact of the supply shock.

### Indirect Costs

**Figure 6:**
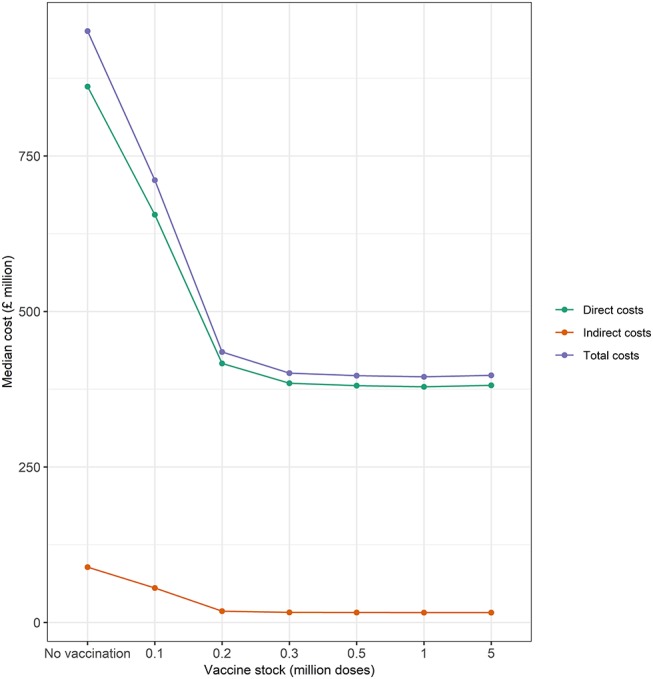
Median direct, indirect, and total costs (£ million) associated with the baseline (no vaccination) and alternative (vaccination) vaccine stock scenarios (i.e., 0.1, 0.2, 0.3, 0.5, 1, and 5 million doses) at the start of the epidemic. Green and orange lines represent median direct and indirect economic costs, respectively.

The magnitude of indirect, direct, and total costs conducive to large outbreaks is presented in Figure 6. These results suggest that economic costs vary with size of the initial vaccine stock. Total economic costs range from £400 to 950 million, with median direct costs between 10 and 24 times larger in magnitude than indirect costs. Indirect costs constitute 9% of total costs under a baseline scenario of no vaccination (i.e., “cull only”) and between 4 and 8% of total costs under alternative scenario of “cull plus vaccinate to live” as the size of the initial vaccine bank decreases from 5 to 0.1 million doses. Losses in revenue in some commodity markets (e.g., beef, pork, lamb, and chicken) are partially offset by gains made in other commodity markets (e.g., milk and feed wheat). Hence, the net effect on indirect costs is likely to be lower compared to the presumption that all commodity markets lose revenue during an outbreak.

**Figure 7:**
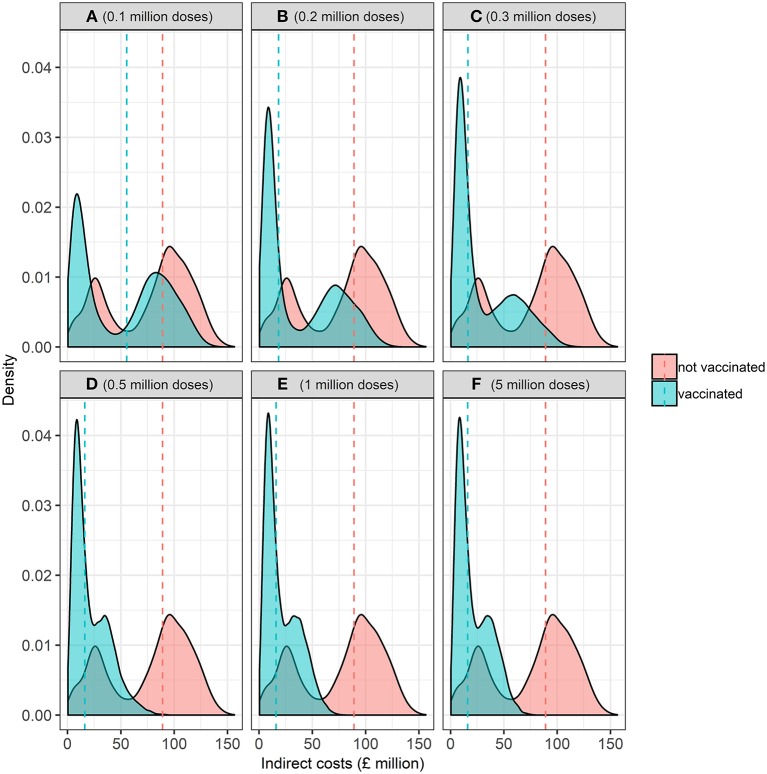
Kernel probability density function of the distribution of indirect economic costs (£ million) associated with the baseline (no vaccination) and alternative (vaccination) vaccine stock scenarios at the start of the epidemic associated with **(A)** 0.1, **(B)** 0.2, **(C)** 0.3, **(D)** 0.5, **(E)** 1, and **(F)** 5 million doses. Dashed vertical red and blue lines represent the median indirect economic costs for the baseline and alternative scenarios, respectively.

The distribution of indirect costs, i.e., the economic losses incurred in markets after disease freedom is declared, in the baseline and alternative vaccine stock scenarios is presented in Figure 7. Controlling an FMD epidemic with vaccination has a lower median indirect cost than the baseline scenario of no vaccination (i.e., “cull only”). Overall, there is less uncertainty, i.e., spread, in indirect costs associated with vaccination compared to the baseline strategy of no vaccination. Varying the size of the vaccine stock impacts on the variability of indirect costs associated with an outbreak. There is wider variation in indirect costs associated with alternative scenario of between 0.1 and 0.3 million compared to 0.5 to 5 million doses in the vaccine bank. These results suggest that vaccination is relatively more beneficial than a strategy of no vaccination. However, more uncertainty is associated with fewer doses of vaccines (i.e., 0.1 to 0.3 million) compared to more doses of vaccines (i.e., 0.5 to 5 million) in the bank, when considering indirect costs.

## Discussion

Our study confirms controlling an FMD epidemic using vaccination costs less, on average, than under a no vaccination strategy (4). In our study, indirect costs constitute only between 4 and 9% of total costs. In other studies indirect costs exceed direct costs, i.e., between 79 and 97% (4) or 29% of total costs (5). Over the last decade the indirect costs of zoonotic disease have contributed 91% of total costs (3). To a certain extent, the ratio of indirect to direct costs may depend on the definition of direct and indirect costs used. Our definition of indirect costs considers indirect costs as the losses after disease freedom is declared and is comprised of change in revenue in various agricultural commodity markets. Economic losses experienced during an outbreak are defined as direct costs. In our study, the loss in revenue in some commodity markets (e.g. beef, pork, lamb, and chicken) are partially offset by gains made in other markets (e.g. milk and feed wheat). Therefore, the overall net effect suggests the magnitude of indirect costs is lower than had all the commodity markets suffered a reduction in revenue. Furthermore, the loss in revenue in exports was not considered because export trade data were not available. The loss of export trade may persist beyond disease freedom should importing countries adopt a precautionary approach. Other examples of offsetting could include: decrease in employment in a particular sector and increase in employment to an economy overall; costs to one farm offset by gains to other farms; and reduced tourism expenditure vs. an expansion in household expenditure (7). Furthermore, an exotic disease, such as FMD, is likely to have indirect consequences that were felt over a larger number of sectors than this study attempts to quantify, e.g. tourism, and retail (7). Lower than expected indirect costs may also be explained by the vaccine bank scenarios considered in this paper because only the worst case scenario for direct costs was evaluated (39). In addition, we have not considered the costs of farm management practices such as restocking livestock, following disease freedom, that could have disease implications but such costs could be included as additional component of indirect costs. In the future, it would be interesting to consider other scenarios and how the trade-off between direct and indirect costs varies under alternative prevention and control strategies.

Economic cost frameworks that classify costs as either direct or indirect are often subjective and there lacks a consistent framework to evaluate them, making it difficult to assess the costs of alternative animal disease relative to one another. An economic cost framework should “(1) Be consistent with economic principles; (2) Be derived from and consistent with veterinary control measures; and (3) Include an explicit definition of the economic perspective and the stakeholders included” (18). Our framework meets these three criteria because; (1) it distinguishes objectively between direct and indirect economic costs using disease status to avoid double counting costs; (2) the estimation of costs is derived from veterinary control measures adopted by the Scottish Government in an epidemic scenario; and (3) direct costs incurred during an outbreak are broken down by economic perspective of government and industry (19) while indirect costs come from the economic perspective of markets considered, in our case the producers of agricultural commodities. This indirect cost framework can be extended to other economic perspectives, e.g., tourism and retail, explicitly taking the perspective of stakeholders beyond the farmgate that are impacted after an outbreak, which is often not considered in other studies. This aspect is important for policy makers responsible for disease outbreak prevention and mitigation decisions who may be required to make important and difficult choices at regional, national or international level. Failure to account for impacts beyond the farming sector has been a criticism of decision making in previous UK FMD epidemics (73, 74). Besides the direct costs associated with production, economic models may be called upon to inform producers and policy-makers of the broader knock-on effects associated with indirect costs after disease freedom is declared because such costs might affect various markets and also have implications for trade.

This paper presents a framework that outlines the necessary steps to estimate indirect costs, i.e., the economic losses incurred in markets after disease freedom is declared, using time series analysis. Using agricultural commodity data, time series models can capture market dynamics and the knock-on price and quantity effects between markets, which are often omitted from traditional farm account-based CBAs and other methods. PE and CGE models have been used to explore the indirect costs of animal disease outbreaks. A drawback of these models is that that they are sometimes based on strong assumptions in the absence of good data, e.g., elasticities, defining the demand and supply relationships in multiple sectors to anticipate the likely economic impact of a supply shock. Often elasticities are taken from the literature or assumed unless estimations are developed directly for the model. By contrast, our econometric approach estimates elasticities directly from data to capture production and price dynamics and equilibrium levels, without the need for relying on the literature or making key assumptions and as such can complement PE or CGE models.

PE models can examine a single sector or multiple markets capturing changes in production and prices. An advantage of CGEs over time series models is that they can represent an entire economy. However, a drawback to CGEs is the use of more complex modeling techniques and results that can be difficult to interpret (21). Our time series model can incorporate further markets and sectors, providing such data are available, without the modeling complexity of a CGE. A review of economic models found that CGEs do not explicitly link to an epidemiological model because this requires further development (21). Multi-market models have also been used to model the impact of changing access to export markets on breeding and investment decisions (24). Such integration does not feature in our time series model. Nevertheless, our time series model is linked with the epidemiological model because the indirect costs are derived from the number of animals culled. Although the models are not integrated fully, a development which requires further interdisciplinary research. Despite this, our paper illustrates the usefulness of time series analysis in modeling the indirect economic costs of an animal disease outbreak.

FMD is not a zoonotic disease i.e., it has no human health or food safety risk. For this reason, the retail response of consumer demand was not considered. However, the FMD outbreak of 2001 had psychological impacts on members of the rural community (75). Indirect costs arising from tourism were also not considered. It is suggested that economic losses arising from the tourism industry are similar in magnitude to that of losses to agriculture and the food supply chain (15). Our framework demonstrates how indirect costs can be estimated, but this study does not quantify all potential indirect costs. The scope of indirect costs can be broadened with our methodology provided that appropriate data, such as tourism revenue and retail market, are available. Our IRF was fit with 12 response variables, each with 156 observations (i.e., 13 years of monthly data), and three shocks but did not have enough forecasting power to include additional variables. To investigate the relationship between the supply shock of animals culled and tourism or consumer demand would require an extension of the data series or fewer impulse or response variables, which was not possible for this study. Alternatively, a separate time series model to represent consumer demand and tourism could be considered.

Data availability can also restrict the scope of indirect costs estimated when considering time series modeling. UK market data are available but often such data are not disaggregated into UK administrations, such that regionalization cannot be accurately considered. For this reason, access to publically available data at a disaggregation required, i.e., monthly observations for Scotland, at the farmgate can be problematic. Where possible we have used Scottish-specific data in our model, otherwise UK-level farmgate price and quantity data were adjusted to reflect a regionalisation for Scotland. Likewise, net export trade data were only available quarterly for the UK-level, however such an aggregation did not allow for sufficient variation in the data. Hence, indirect costs do not capture the impact of animal disease outbreak on trade flows. If a country affected by a disease outbreak is a large exporter of livestock, a shock in the domestic market will have knock-on effects into international markets, and consequently international prices if there is an export ban (76).

If animal health is considered a public good (77), better estimates of indirect costs are necessary to support decision-making for animal disease prevention and control strategies.

The Scottish Government's animal health and welfare strategy is to prioritize limited resources and to consider cost sharing responsibilities for preventing and controlling disease. Therefore, indirect costs are a concern for policy makers to understand the cost of alternative prevention and control strategies in context of one another given the allocation of limited resources. Governments need to appropriately balance the costs of disease control between industry and the tax payer, ensuring financial support for farmers and value for money for taxpayers. During the 2001 UK FMD outbreak, farmers were compensated £1.4 billion for the slaughter of animals and disposal and clean-up costs. In addition, the epidemic costed £1.3 billion to eradicate and other public sector costs amounted to £0.3 billion. The private sector was not compensated but also experienced losses; agriculture, food supply chain and supporting services lost £0.6 billion, while the outbreak costed tourism and supporting industries between £4.5–5.4 billion (41). The moral hazard problem arises when not all stakeholders are compensated (78, 79). When compensation is expected it may create incentives for individuals to act in ways that incur costs that they know they will not have to bear. Compensation must be large enough to ensure reporting of disease but not so large to discourage preventative biosecurity (78). Partial compensation helps spread some of the risk responsibility to farmers. Nevertheless, all those that incur losses of an epidemic are not compensated. Therefore, determining how indirect costs are distributed helps address this by informing government of stakeholders, besides farmers and the farming industry, that are impacted by an outbreak and should potentially be considered for compensation after an outbreak is over.

The indirect cost methodology presented in this study is applicable not only to FMD but also other exotic animal diseases. Furthermore, the method is particularly pertinent in light of Brexit, which can be thought of as a “shock” that may alter the UK's livestock disease risk and disrupt markets. It will be important to evaluate the knock-on-indirect effects of alternative Brexit scenarios that are likely to arise from changes to trade rules and access to pharmaceuticals. New trading arrangements may affect the import and export of livestock products and also the movement of animals which may alter the UK's disease risk and interrupt the supply chain. In the context of changing trade relationships with global trading partners under Brexit, understanding indirect costs will become even more important. The UK is a net importer of agri-food production from the EU, which could have implications for EU farming and food sectors (80). The anticipated price and production changes will vary depending on trade agreement considered and whether the UK is a net importer or export of individual commodities concerned (81). There are also concerns as to the supply and access of vaccines post-Brexit (82). In an emergency epidemic, this could pose a risk to the UK's disease status, food security and could have knock-on effects for trade relationships. For example, the UK may no longer have access to the European Union's FMD vaccine bank. The UK has a reference laboratory for FMD but in the face of an outbreak would the UK's national vaccine bank have sufficient stock? Hence, as much uncertainty remains, it is important that alternative Brexit scenarios are considered to anticipate the perceived impact of leaving the EU on various agricultural markets and rural sectors of the economy and the implications for disease risk and the associated economic costs.

## Conclusion

In conclusion, this paper has presented a framework for defining and estimating the indirect costs, i.e., the economic losses incurred in markets after disease freedom is declared, of an animal disease outbreak. The time series model identified was a VECM, a useful tool for capturing knock-on market dynamics following a disease freedom/outbreak. Overall, in terms of indirect costs it is more beneficial to vaccinate compared to a “cull only” FMD control strategy. Our findings suggest indirect costs vary with the size of the initial vaccine stock and are less variable when vaccination is used instead of culling. The estimation of indirect costs contributes to the overall economic assessment of the costs of an animal disease outbreak, which is often overlooked but is necessary in support of decision-making. In future, constraints on data and analytical frameworks that otherwise limit the estimation of indirect costs should be addressed. The framework presented can be applied to other animal disease scenarios to more consistently evaluate indirect costs. It is important that indirect costs are not overlooked because their estimation is necessary for a more complete picture of the costs of animal disease outbreaks across case studies to better prioritize limited resources and inform cost sharing. Our indirect cost modeling framework can be adapted to model how changes in the political economy, such as Brexit, might impact the cost of animal disease outbreaks in the future.

## Author Contributions

AS developed the initial concept with GG, Dr. Habtu Weldegebriel, and TP, with further refinement from AB and KR. AB gathered data, developed the indirect cost model with KR and JE and carried out the modeling. AB drafted the paper, and KR, JE, AS, TP, and GG also contributed to the manuscript. GG and AS secured funding and led the project. All authors gave final approval for publication.

### Conflict of Interest Statement

The authors declare that the research was conducted in the absence of any commercial or financial relationships that could be construed as a potential conflict of interest. The handling editor is currently editing and co-organizing a Research Topic with one of the authors GG, and confirms the absence of any other collaboration.

## Footnotes

^1^Elasticity measures the extent to which a proportional change in one variable is associated with a proportional change in another variable.
