# Supplementary material for: Framework for Estimating Indirect Costs in Animal Health Using Time Series Analysis
Source: Front Vet Sci. 2019 Jun 18;6:190. doi: 10.3389/fvets.2019.00190 (PMC6592220; doi:10.3389/fvets.2019.00190)
Supplement: Supplementary file 1 [file Data_Sheet_1.PDF]

Decomposed data series − Real producer price of beef

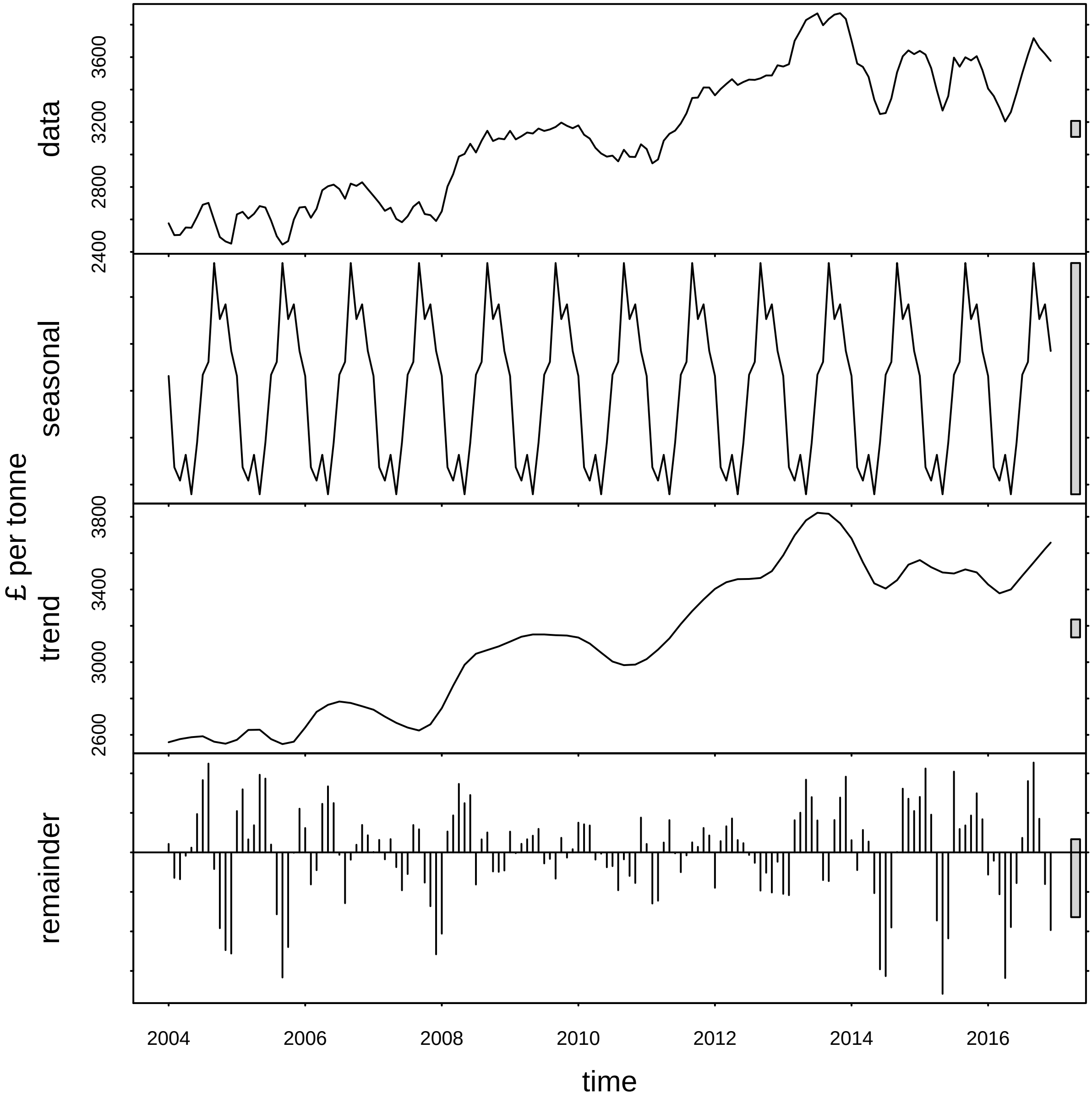

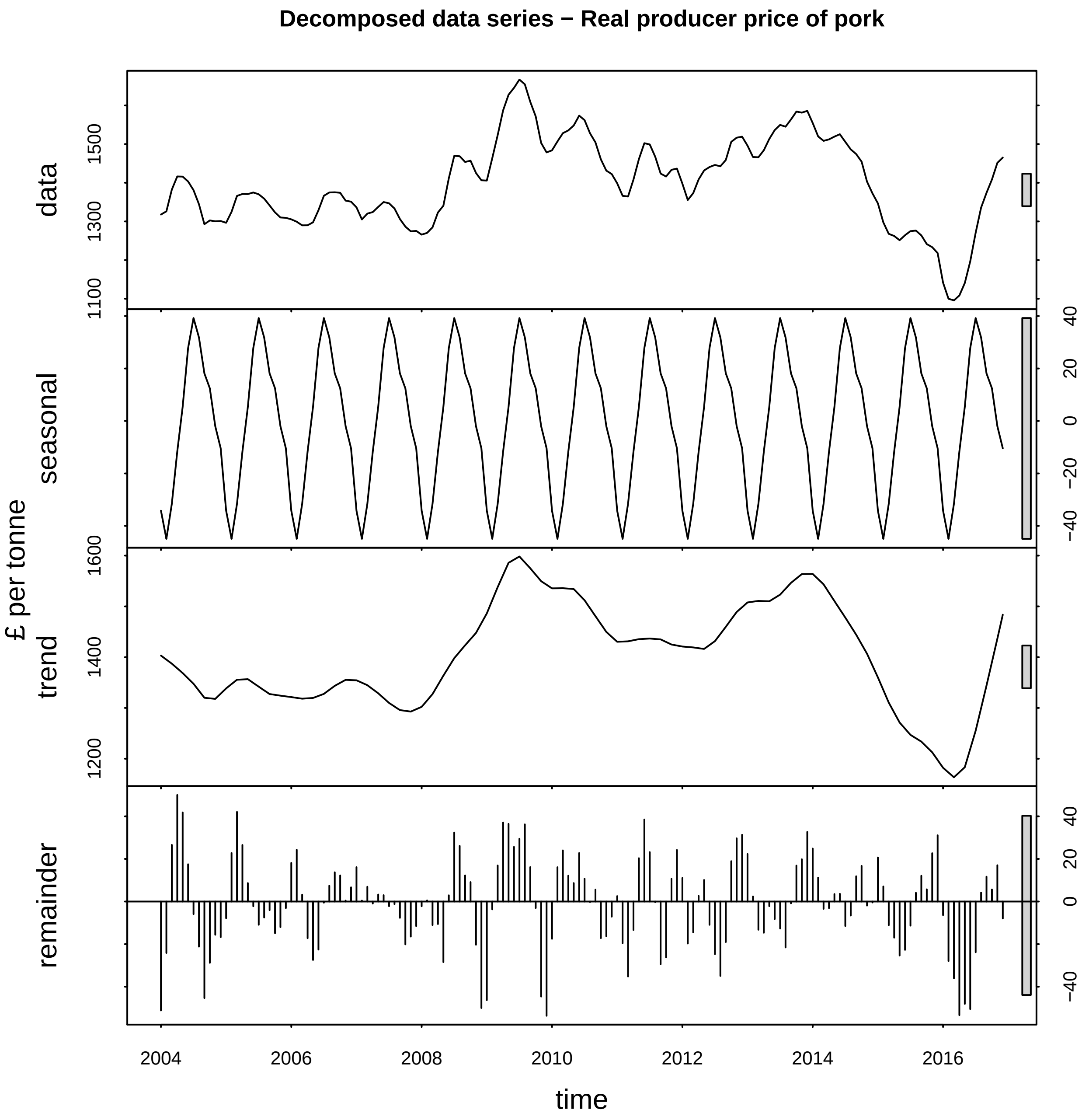

Decomposed data series − Real producer price of pork
data
seasonal
trend
remainder
£ per tonne
time
1100
1300
1500
40
20
0
−20
−40
1200
1400
1600
40
20
0
−40
2004
2006
2008
2010
2012
2014
2016

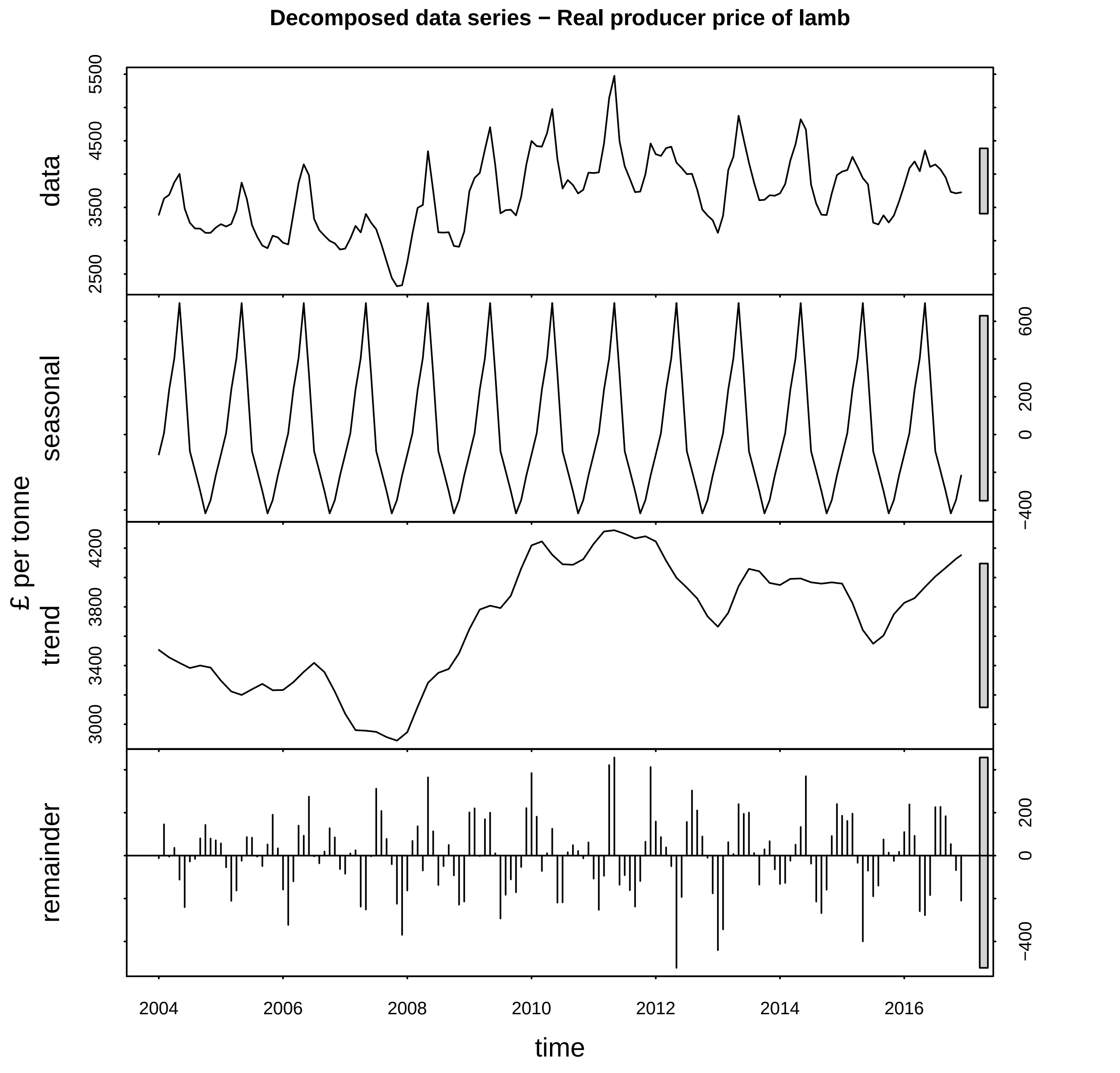

Decomposed data series − Real producer price of lamb
data
seasonal
trend
remainder
£ per tonne
time
2500
3500
4500
5500
−400
0
200
600
3000
3400
3800
4200
−400
0
200
2004
2006
2008
2010
2012
2014
2016

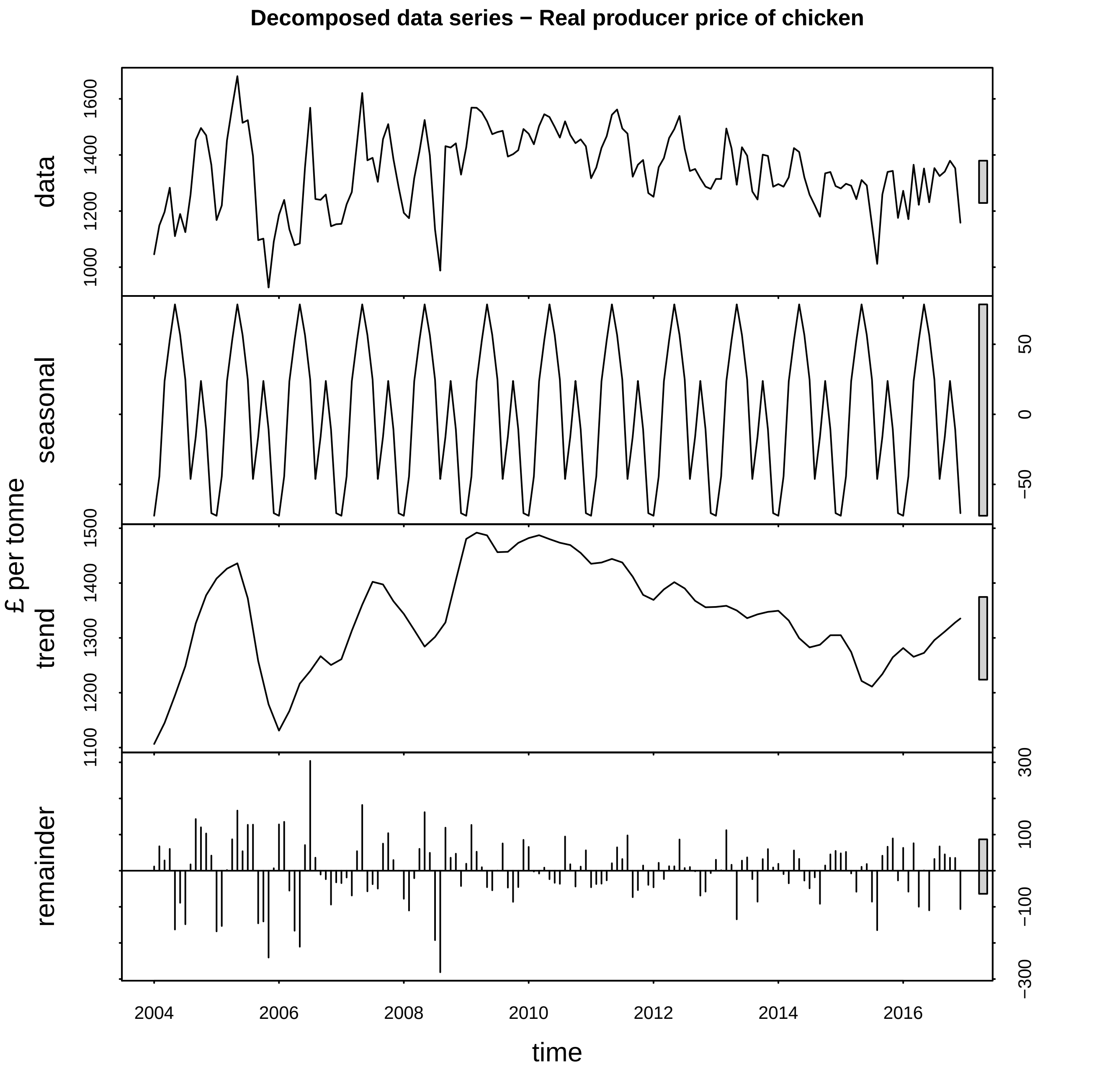

Decomposed data series − Real producer price of chicken
data
1000
1200
1400
1600
seasonal
−50
0
50
£ per tonne
trend
1100
1200
1300
1400
1500
remainder
−300
−100
100
300
2004
2006
2008
2010
2012
2014
2016
time

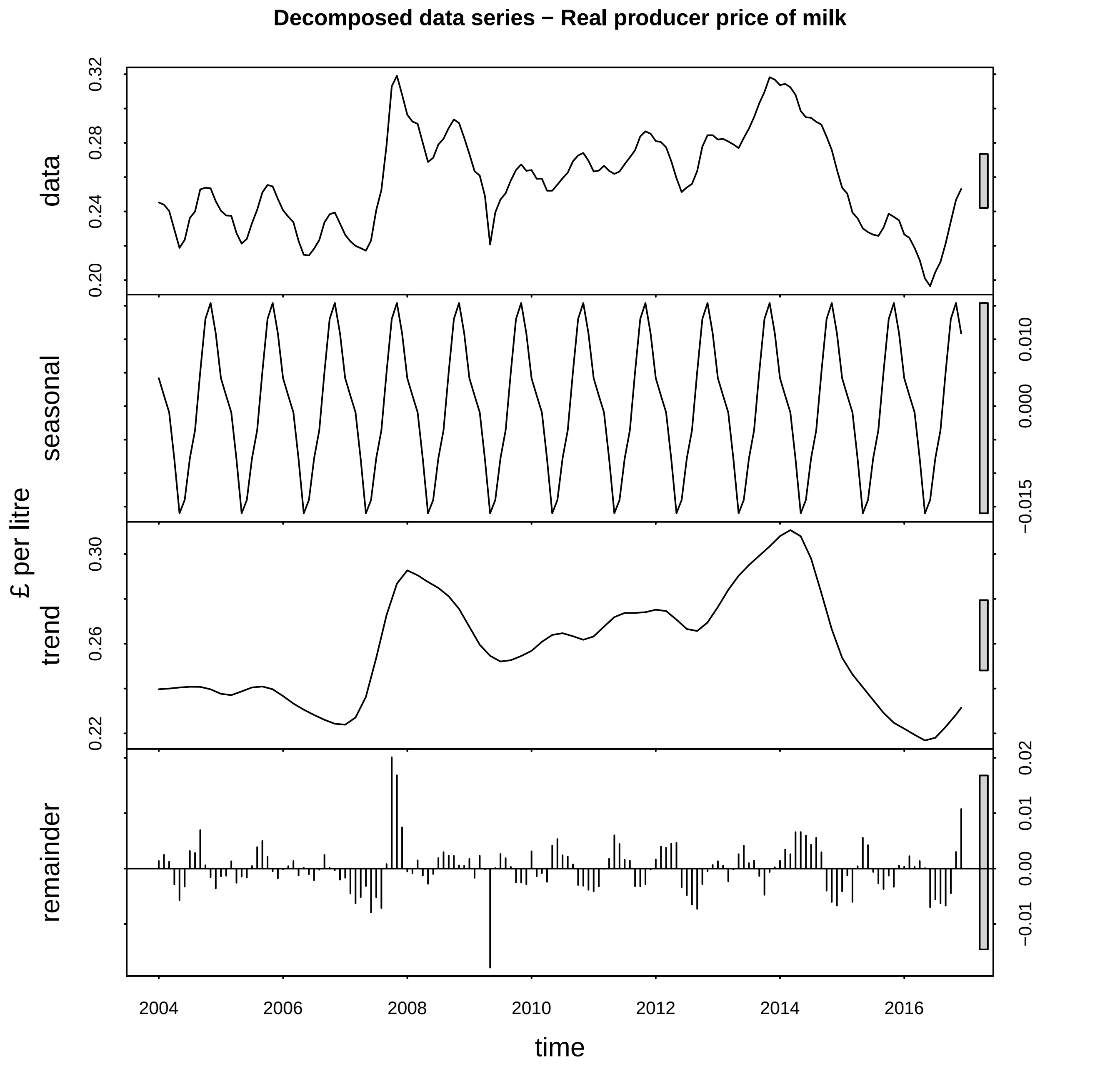
Decomposed data series − Real producer price of milk
data
seasonal
trend
remainder
£ per litre
time
0.20
0.24
0.28
0.32
−0.015
0.000
0.010
0.22
0.26
0.30
−0.01
0.00
0.01
0.02
2004
2006
2008
2010
2012
2014
2016

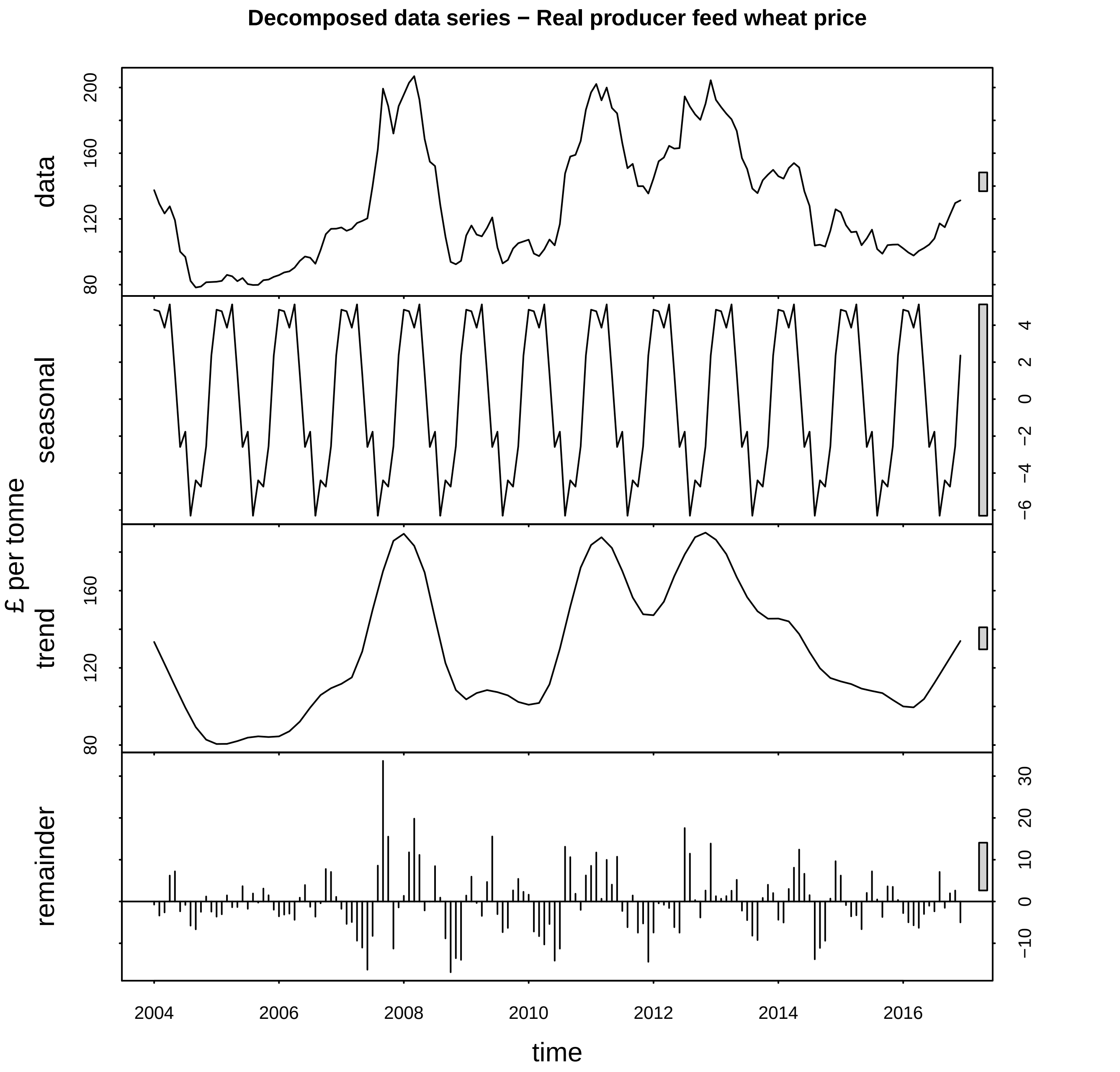

Decomposed data series − Real producer feed wheat price
data
seasonal
trend
remainder
£ per tonne
time
200
160
120
80
4
2
0
−2
−4
−6
160
120
80
30
20
10
0
−10
2004
2006
2008
2010
2012
2014
2016

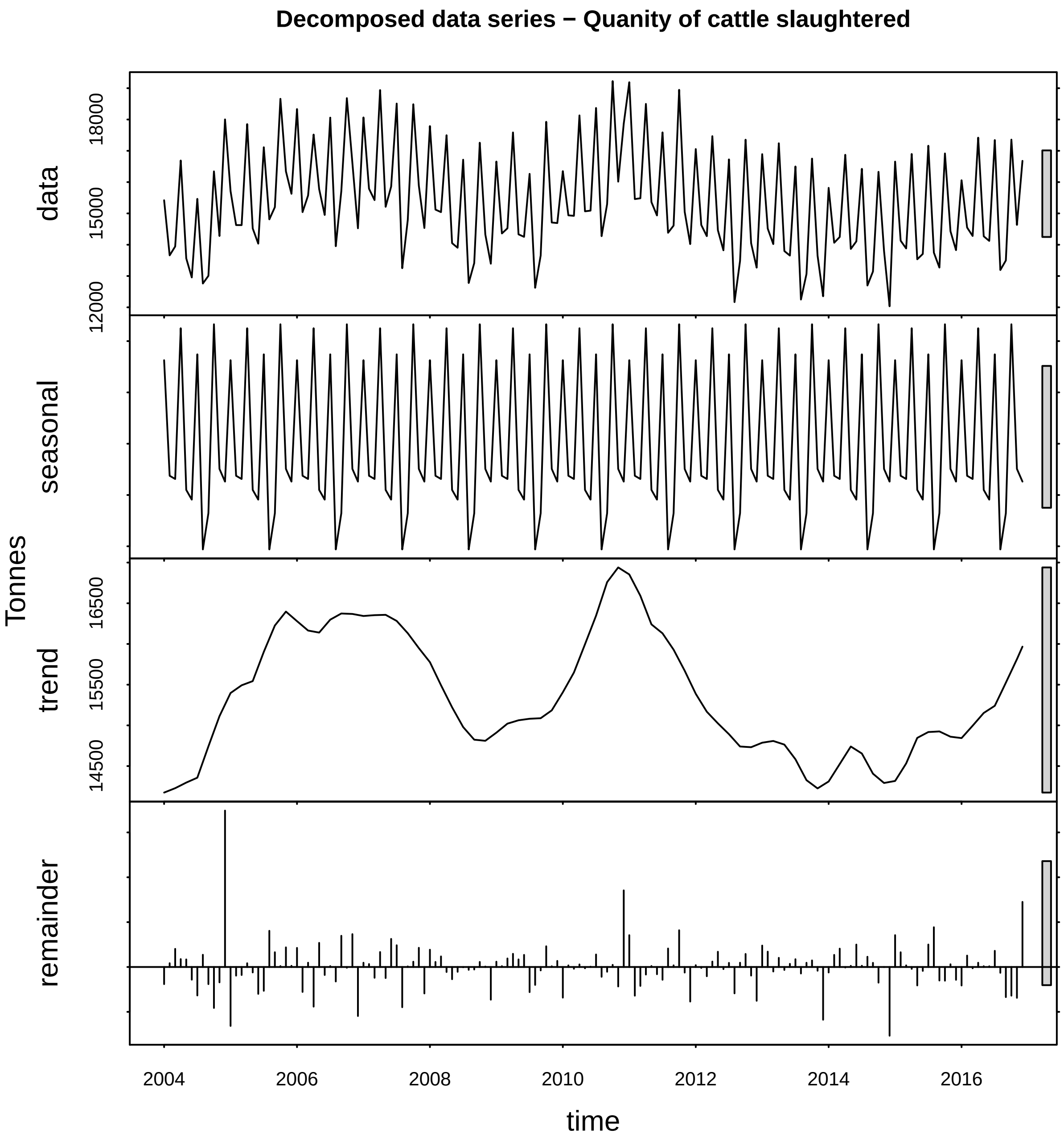

Decomposed data series − Quanity of cattle slaughtered
data
seasonal
trend
remainder
Tonnes
time
12000
15000
18000
−2000
0
1000
14500
15500
16500
−1000
1000
3000
2004
2006
2008
2010
2012
2014
2016

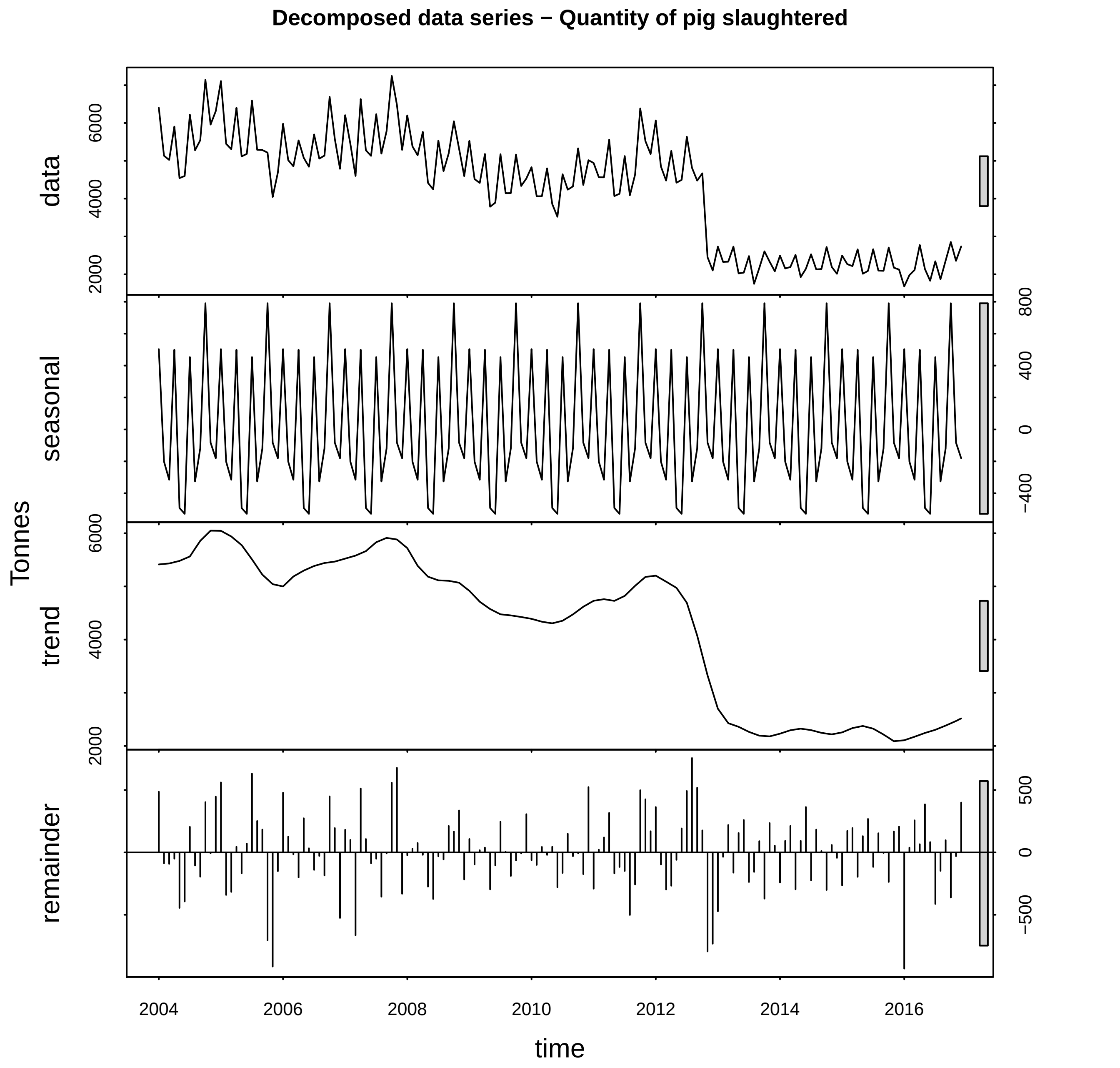
Decomposed data series − Quantity of pig slaughtered
data
seasonal
trend
remainder
Tonnes
time
2000
4000
6000
800
400
0
−400
500
−500
2004
2006
2008
2010
2012
2014
2016

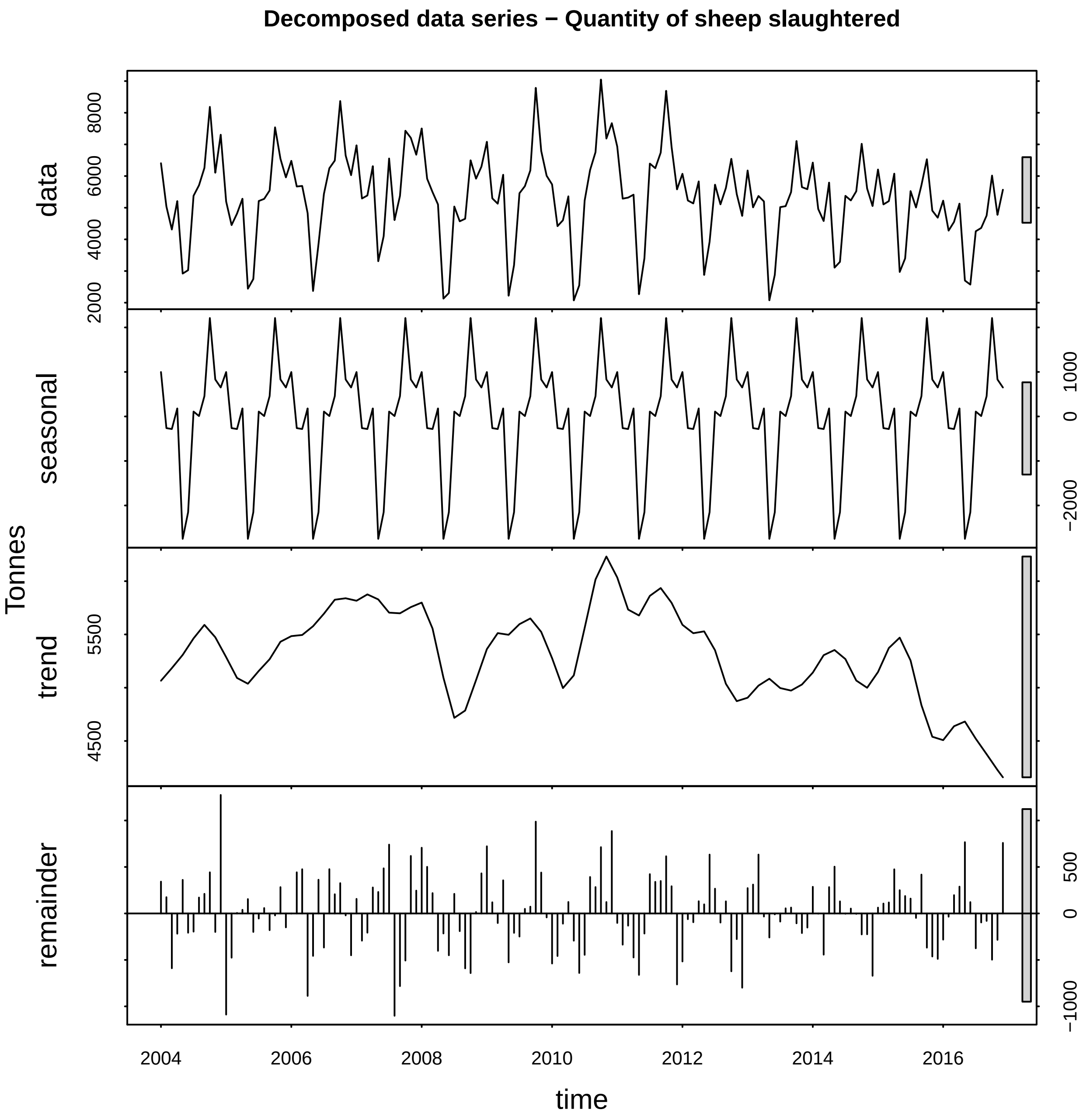

Decomposed data series − Quantity of sheep slaughtered
data
2000
4000
6000
8000
seasonal
−2000
0
1000
Tonnes
trend
4500
5500
remainder
−1000
0
500
2004
2006
2008
2010
2012
2014
2016
time

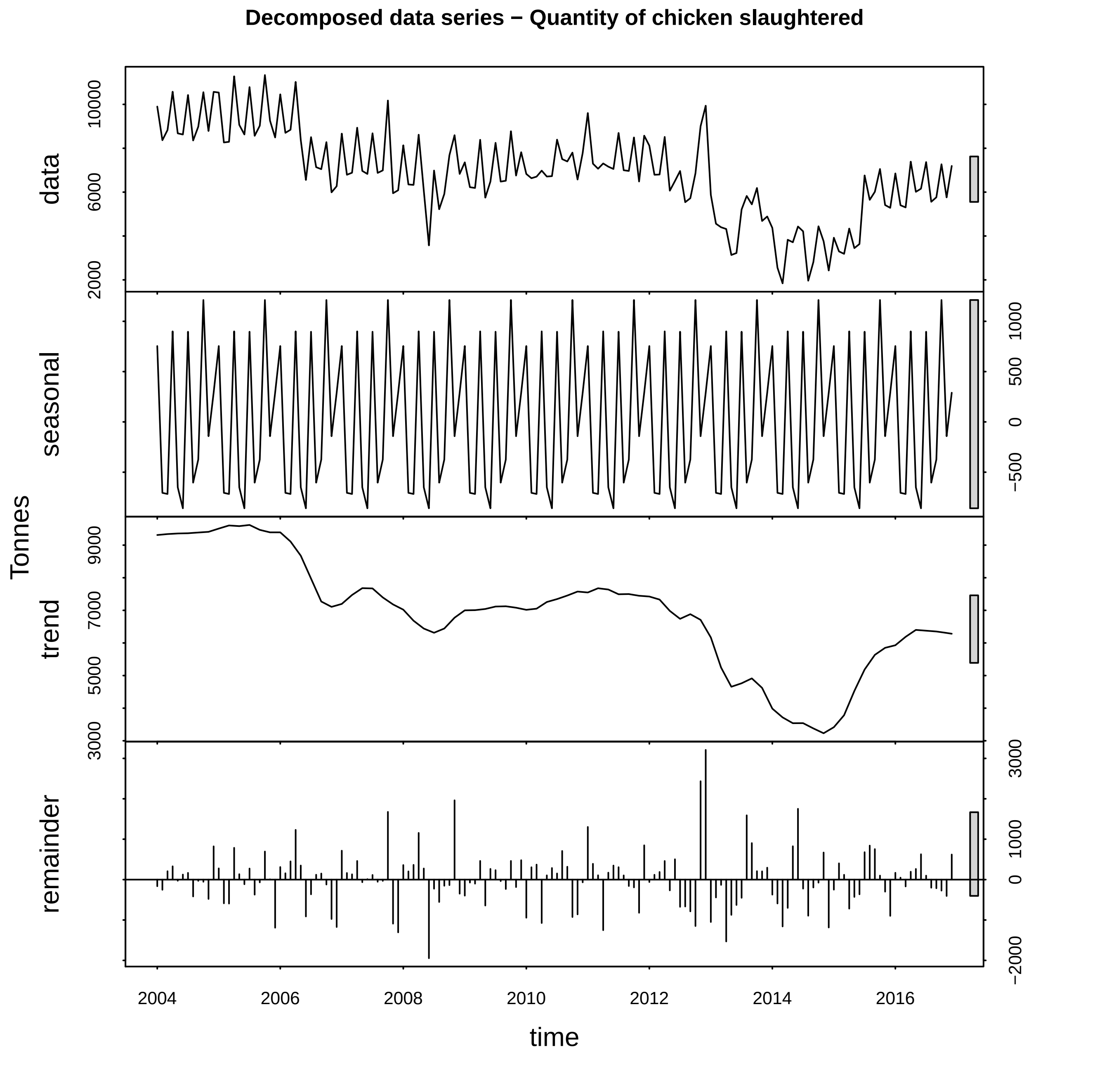
Decomposed data series − Quantity of chicken slaughtered
data
seasonal
trend
remainder
Tonnes
10000
6000
2000
1000
500
0
−500
9000
7000
5000
3000
3000
1000
0
−2000
2004
2006
2008
2010
2012
2014
2016
time

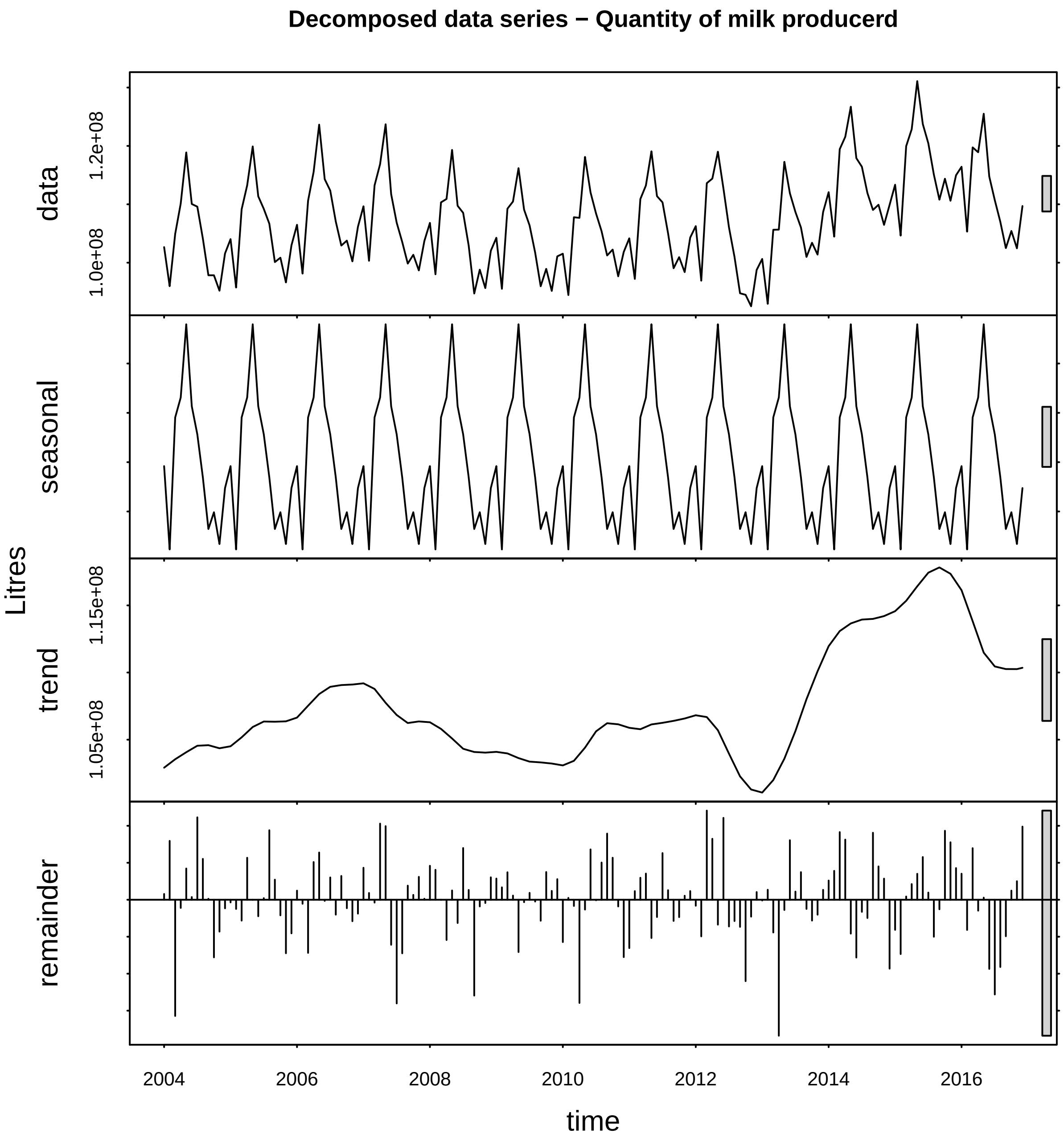

Decomposed data series − Quantity of milk producerd
data
1.0e+08
1.2e+08
seasonal
−5e+06
5e+06
trend
1.05e+08
1.15e+08
remainder
−3e+06
0e+00
2e+06
Litres
2004
2006
2008
2010
2012
2014
2016
time

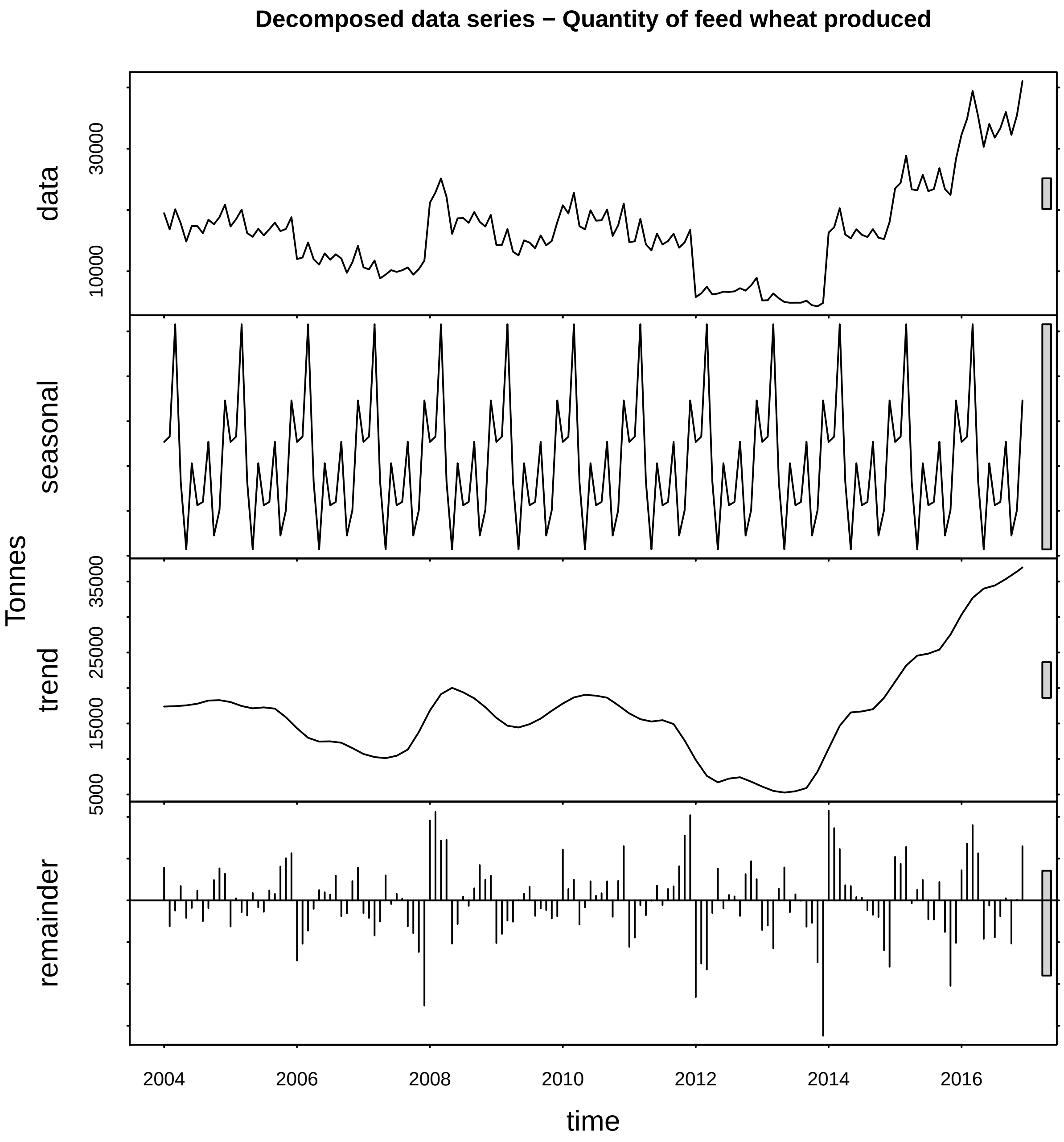
Decomposed data series − Quantity of feed wheat produced
data
seasonal
trend
remainder
Tonnes
time
10000
30000
−2000
0
1000
3000
5000
15000
25000
35000
−6000
−2000
2000
2004
2006
2008
2010
2012
2014
2016
