# Supplementary material for: Framework for Estimating Indirect Costs in Animal Health Using Time Series Analysis
Source: Front Vet Sci. 2019 Jun 18;6:190. doi: 10.3389/fvets.2019.00190 (PMC6592220; doi:10.3389/fvets.2019.00190)
Supplement: Supplementary file 2 [file Data_Sheet_2.PDF]

Table S1. Vector error correction model intercept estimates, μ_0, for each cointegrating equation

Cointegrating equation	Intercept coefficient, μ_0
Δ In $PBeef_t$	0.293
Δ In $PPork_t$	0.887
Δ In $PLamb_t$	-3.551
Δ In $PChicken_t$	8.112
Δ In $PMilk_t$	-1.246
Δ In $PWheat_t$	-3.863
Δ In $QBeef_t$	-0.431
Δ In $QPork_t$	-0.196
Δ In $QSheep_t$	-5.839
Δ In $QChicken_t$	20.808
Δ In $QMilk_t$	0.549
Δ In $QWheat_t$	-5.283

Maximum likelihood estimation
AIC= -11,027.32, BIC= -10,419.93
N=156
Lag=1
Rank=2
Constant deterministic regressor

Table S2. Vector error correction model short-run dynamic adjustment coefficient matrix, Γ_i

Cointegrating equation	$\Delta \ln PBeef_{t-1}$	$\Delta \ln PPork_{t-1}$	$\Delta \ln PLamb_{t-1}$	$\Delta \ln PChicken_{t-1}$	$\Delta \ln PMilk_{t-1}$	$\Delta \ln PWheat_{t-1}$	$\Delta \ln QBeef_{t-1}$	$\Delta \ln QPork_{t-1}$	$\Delta \ln QSheep_{t-1}$	$\Delta \ln QChicken_{t-1}$	$\Delta \ln QMilk_{t-1}$	$\Delta \ln QWheat_{t-1}$
$\Delta \ln PBeef_t$	-0.01 (0.018)	0.293 (0.595)	0.409 (0.087) ***	0.089 (0.098)	0.02 (0.032)	-0.053 (0.022) *	-0.075 (0.085)	-0.009 (0.025)	0.004 (0.036)	-0.015 (0.012)	0.027 (0.02)	0.007 (0.01)
$\Delta \ln PPork_t$	-0.04 (0.012) ***	0.887 (0.38) *	0.032 (0.055)	0.725 (0.062) ***	0.047 (0.02) *	0.019 (0.014)	0.121 (0.055) *	0.005 (0.016)	-0.002 (0.023)	-0.006 (0.008)	-0.022 (0.012) .	-0.006 (0.006)
$\Delta \ln PLamb_t$	0.049 (0.051)	-3.551 (1.649) *	0.312 (0.241)	-0.447 (0.27)	0.142 (0.088)	-0.097 (0.06)	-0.449 (0.237) .	-0.068 (0.068)	-0.05 (0.1)	0.032 (0.034)	0.051 (0.054)	-0.008 (0.028)
$\Delta \ln PChicken_t$	0.12 (0.071) .	8.112 (2.303) ***	0.19 (0.336)	-0.665 (0.378) .	-0.01 (0.123)	0.12 (0.084)	0.132 (0.331)	0.111 (0.096)	-0.003 (0.139)	0.04 (0.048)	0.048 (0.076)	-0.011 (0.039)
$\Delta \ln PMilk_t$	0.054 (0.017) **	-1.246 (0.553) *	-0.103 (0.081)	-0.038 (0.091)	0.033 (0.03)	0.009 (0.02)	0.306 (0.079) ***	0.053 (0.023) *	0.051 (0.033)	-0.007 (0.012)	-0.036 (0.018) .	-0.023 (0.009) *
$\Delta \ln PWheat_t$	-0.022 (0.059)	-3.863 (1.926) *	-0.079 (0.281)	0.091 (0.316)	0.103 (0.103)	-0.08 (0.07)	-0.442 (0.277)	0.325 (0.08) ***	0.045 (0.116)	-0.004 (0.04)	-0.045 (0.063)	-0.015 (0.032)
$\Delta \ln QBeef_t$	0.003 (0.045)	-0.431 (1.453)	-0.461 (0.212) *	0.167 (0.238)	0.024 (0.078)	0.011 (0.053)	-0.09 (0.209)	0.05 (0.06)	-0.443 (0.088) ***	-0.022 (0.03)	-0.036 (0.048)	-0.036 (0.024)
$\Delta \ln QPork_t$	-0.021 (0.12)	-0.196 (3.914)	0.115 (0.572)	-1.282 (0.642) *	-0.307 (0.209)	0.176 (0.143)	0.282 (0.562)	0.152 (0.162)	0.283 (0.236)	-0.296 (0.082) ***	-0.035 (0.128)	0.025 (0.065)
$\Delta \ln QSheep_t$	0.308 (0.086) ***	-5.839 (2.802) *	-0.682 (0.409) .	-0.732 (0.459)	-0.489 (0.15) **	0.023 (0.102)	-0.252 (0.402)	0.043 (0.116)	-0.253 (0.169)	0.044 (0.059)	-0.152 (0.092)	-0.079 (0.047) .
$\Delta \ln QChicken_t$	-0.62 (0.143) ***	20.808 (4.647) ***	-0.515 (0.679)	1.885 (0.762) *	-0.607 (0.248) *	0.199 (0.169)	1.101 (0.667)	0.318 (0.193)	0.086 (0.28)	-0.23 (0.097) *	-0.143 (0.152)	-0.081 (0.078)
$\Delta \ln QMilk_t$	0.004 (0.013)	0.549 (0.415)	0.002 (0.061)	0.011 (0.068)	-0.053 (0.022) *	0.013 (0.015)	0.058 (0.06)	0.015 (0.017)	-0.015 (0.025)	0.013 (0.009)	-0.013 (0.014)	-0.009 (0.007)
$\Delta \ln QWheat_t$	-0.117 (0.201)	-5.283 (6.557)	-0.622 (0.958)	0.621 (1.075)	0.178 (0.35)	-0.228 (0.239)	-0.267 (0.942)	0.003 (0.272)	-0.36 (0.396)	-0.102 (0.137)	0.183 (0.215)	-0.146 (0.11)

Standard error in parentheses
. $p<0.1$, * $p\leq0.05$, ** $p\leq0.01$, *** $p\leq0.001$
Maximum likelihood estimation
AIC= -11,027.32, BIC= -10,419.93
N=156
Lag=1
Rank=2
Constant deterministic regressor

Table S3. Vector error correction model error adjustment coefficients estimates, α_1 and α_2, for the error correction terms of each cointegrating equation.

Cointegrating equation	α_1	α_2
Δ In $PBeef_t$	0.000	-0.010
Δ In $PPork_t$	-0.001	-0.040
Δ In $PLamb_t$	-0.023	0.049
Δ In $PChicken_t$	0.111	0.120
Δ In $PMilk_t$	0.002	0.054
Δ In $PWheat_t$	-0.044	-0.022
Δ In $QBeef_t$	-0.003	0.003
Δ In $QPork_t$	-0.007	-0.021
Δ In $QSheep_t$	0.021	0.308
Δ In $QChicken_t$	0.047	-0.620
Δ In $QMilk_t$	0.007	0.004
Δ In $QWheat_t$	-0.082	-0.117

Maximum likelihood estimation
AIC= -11,027.32, BIC= -10,419.93
N=156
Lag=1
Rank=2
Constant deterministic regressor

Table S4. Vector error correction model long-run adjustment cointegrating error correction coefficients, β_1 and β_2, for each cointegrating equation.

Cointegrating equation	β_1	β_2
Δ In $PBeef_t$	1	0
Δ In $PPork_t$	0	1
Δ In $PLamb_t$	0.999	-0.444
Δ In $PChicken_t$	-4.119	0.183
Δ In $PMilk_t$	0.551	-0.206
Δ In $PWheat_t$	-0.144	0.223
Δ In $QBeef_t$	-0.134	0.251
Δ In $QPork_t$	0.566	-0.087
Δ In $QSheep_t$	-0.310	-0.636
Δ In $QChicken_t$	-0.474	0.314
Δ In $QMilk_t$	-4.601	1.126
Δ In $QWheat_t$	0.231	-0.019

Maximum likelihood estimation
AIC= -11,027.32, BIC= -10,419.93
N=156
Lag=1
Rank=2
Constant deterministic regressor

Table S5. Vector error correction model error correction matrix, ∏.

Cointegrating equation	Δ ln $PBeef_{t-1}$	Δ ln $PPork_{t-1}$	Δ ln $PLamb_{t-1}$	Δ ln $PChicken_{t-1}$	Δ ln $PMilk_{t-1}$	Δ ln $PWheat_{t-1}$	Δ ln $QBeef_{t-1}$	Δ ln $QPork_{t-1}$	Δ ln $QSheep_{t-1}$	Δ ln $QChicken_{t-1}$	Δ ln $QMilk_{t-1}$	Δ ln $QWheat_{t-1}$
Δ ln $PBeef_t$	0.000	-0.010	0.005	-0.003	0.002	-0.002	-0.003	0.001	0.007	-0.003	-0.013	0.000
Δ ln $PPork_t$	-0.001	-0.040	0.016	-0.001	0.007	-0.009	-0.010	0.003	0.026	-0.012	-0.038	0.000
Δ ln $PLamb_t$	-0.023	0.049	-0.044	0.102	-0.023	0.014	0.015	-0.017	-0.024	0.026	0.159	-0.006
Δ ln $PChicken_t$	0.111	0.120	0.058	-0.435	0.036	0.011	0.015	0.052	-0.111	-0.015	-0.375	0.023
Δ ln $PMilk_t$	0.002	0.054	-0.022	0.003	-0.010	0.012	0.013	-0.004	-0.035	0.016	0.054	-0.001
Δ ln $PWheat_t$	-0.044	-0.022	-0.034	0.176	-0.020	0.001	0.000	-0.023	0.027	0.014	0.177	-0.010
Δ ln $QBeef_t$	-0.003	0.003	-0.005	0.015	-0.003	0.001	0.001	-0.002	-0.001	0.003	0.020	-0.001
Δ ln $QPork_t$	-0.007	-0.021	0.002	0.026	0.000	-0.004	-0.004	-0.002	0.016	-0.003	0.010	-0.001
Δ ln $QSheep_t$	0.021	0.308	-0.116	-0.031	-0.052	0.066	0.075	-0.015	-0.203	0.087	0.249	-0.001
Δ ln $QChicken_t$	0.047	-0.620	0.322	-0.306	0.154	-0.145	-0.162	0.080	0.380	-0.217	-0.914	0.023
Δ ln $QMilk_t$	0.007	0.004	0.005	-0.026	0.003	0.000	0.000	0.003	-0.005	-0.002	-0.025	0.001
Δ ln $QWheat_t$	-0.082	-0.117	-0.030	0.318	-0.021	-0.014	-0.018	-0.036	0.100	0.002	0.247	-0.017

Maximum likelihood estimation
AIC= -11,027.32, BIC= -10,419.93
N=156
Lag=1
Rank=2
Constant deterministic regressor
