# Supplementary material for: Framework for Estimating Indirect Costs in Animal Health Using Time Series Analysis
Source: Front Vet Sci. 2019 Jun 18;6:190. doi: 10.3389/fvets.2019.00190 (PMC6592220; doi:10.3389/fvets.2019.00190)
Supplement: Supplementary file 3 [file Data_Sheet_3.PDF]

Table S6. Correlation coefficients of variance covariance matrix of cointegrating equations. Where α_1 and α_2 are error correction terms and μ_0 are intercepts

Cointegrating equations	Cointegrating equations / Coefficients	Δ ln PBeef$_t$														
		α_1	α_2	μ_0	ln PBeef$_{t-1}$	ln PPork$_{t-1}$	ln PLamb$_{t-1}$	ln PChicken$_{t-1}$	ln PMilk$_{t-1}$	ln PWheat$_{t-1}$	ln QCattle$_{t-1}$	ln QPig$_{t-1}$	ln QSheep$_{t-1}$	ln QChicken$_{t-1}$	ln QMilk$_{t-1}$	ln QWheat$_{t-1}$
Δ ln PBeef$_t$	α_1	1	0.268	0.642	-0.307	-0.133	0.002	0.442	-0.011	0.285	0.002	-0.016	0.152	0.012	-0.004	0.001
	α_2	0.268	1	-0.567	-0.134	-0.284	0.204	0.024	-0.358	-0.021	0.018	0.172	0.195	-0.190	-0.138	0.099
	μ_0	0.642	-0.567	1	-0.157	0.112	-0.161	0.359	0.276	0.260	-0.013	-0.150	-0.026	0.161	0.107	-0.078
	ln PBeef$_{t-1}$	-0.307	-0.134	-0.157	1	-0.159	-0.265	-0.141	0.158	-0.117	0.015	-0.053	0.114	0.067	-0.081	0.050
	ln PPork$_{t-1}$	-0.133	-0.284	0.112	-0.159	1	0.098	-0.054	0.039	-0.052	-0.118	0.026	0.080	0.010	0.082	-0.023
	ln PLamb$_{t-1}$	0.002	0.204	-0.161	-0.265	0.098	1	-0.099	0.009	-0.182	-0.027	-0.010	0.094	-0.105	0.065	-0.036
	ln PChicken$_{t-1}$	0.442	0.024	0.359	-0.141	-0.054	-0.099	1	0.000	0.188	0.042	0.007	-0.040	0.051	0.000	0.036
	ln PMilk$_{t-1}$	-0.011	-0.358	0.276	0.158	0.039	0.009	0.000	1	-0.069	0.002	-0.168	-0.038	0.079	-0.060	-0.085
	ln PWheat$_{t-1}$	0.285	-0.021	0.260	-0.117	-0.052	-0.182	0.188	-0.069	1	0.011	0.015	-0.015	0.022	0.035	0.022
	ln QCattle$_{t-1}$	0.002	0.018	-0.013	0.015	-0.118	-0.027	0.042	0.002	0.011	1	-0.071	-0.473	-0.087	0.086	-0.123
	ln QPig$_{t-1}$	-0.016	0.172	-0.150	-0.053	0.026	-0.010	0.007	-0.168	0.015	-0.071	1	-0.054	-0.067	-0.011	0.140
	ln QSheep$_{t-1}$	0.152	0.195	-0.026	0.114	0.080	0.094	-0.040	-0.038	-0.015	-0.473	-0.054	1	-0.033	-0.155	0.072
	ln QChicken$_{t-1}$	0.012	-0.190	0.161	0.067	0.010	-0.105	0.051	0.079	0.022	-0.087	-0.067	-0.033	1	-0.161	-0.007
	ln QMilk$_{t-1}$	-0.004	-0.138	0.107	-0.081	0.082	0.065	0.000	-0.060	0.035	0.086	-0.011	-0.155	-0.161	1	0.055
	ln QWheat$_{t-1}$	0.001	0.099	-0.078	0.050	-0.023	-0.036	0.036	-0.085	0.022	-0.123	0.140	0.072	-0.007	0.055	1
Δ ln PPork$_t$	α_1	0.204	0.055	0.131	-0.063	-0.027	0.000	0.090	-0.002	0.058	0.000	-0.003	0.031	0.002	-0.001	0.000
	α_2	0.055	0.204	-0.116	-0.027	-0.058	0.042	0.005	-0.073	-0.004	0.004	0.035	0.040	-0.039	-0.028	0.020
	μ_0	0.131	-0.116	0.204	-0.032	0.023	-0.033	0.073	0.056	0.053	-0.003	-0.031	-0.005	0.033	0.022	-0.016
	ln PBeef$_{t-1}$	-0.063	-0.027	-0.032	0.204	-0.032	-0.054	-0.029	0.032	-0.024	0.003	-0.011	0.023	0.014	-0.016	0.010
	ln PPork$_{t-1}$	-0.027	-0.058	0.023	-0.032	0.204	0.020	-0.011	0.008	-0.011	-0.024	0.005	0.016	0.002	0.017	-0.005
	ln PLamb$_{t-1}$	0.000	0.042	-0.033	-0.054	0.020	0.204	-0.020	0.002	-0.037	-0.005	-0.002	0.019	-0.021	0.013	-0.007
	ln PChicken$_{t-1}$	0.090	0.005	0.073	-0.029	-0.011	-0.020	0.204	0.000	0.038	0.009	0.001	-0.008	0.010	0.000	0.007
	ln PMilk$_{t-1}$	-0.002	-0.073	0.056	0.032	0.008	0.002	0.000	0.204	-0.014	0.000	-0.034	-0.008	0.016	-0.012	-0.017
	ln PWheat$_{t-1}$	0.058	-0.004	0.053	-0.024	-0.011	-0.037	0.038	-0.014	0.204	0.002	0.003	-0.003	0.004	0.007	0.004
	ln QCattle$_{t-1}$	0.000	0.004	-0.003	0.003	-0.024	-0.005	0.009	0.000	0.002	0.204	-0.014	-0.097	-0.018	0.017	-0.025
	ln QPig$_{t-1}$	-0.003	0.035	-0.031	-0.011	0.005	-0.002	0.001	-0.034	0.003	-0.014	0.204	-0.011	-0.014	-0.002	0.029
	ln QSheep$_{t-1}$	0.031	0.040	-0.005	0.023	0.016	0.019	-0.008	-0.008	-0.003	-0.097	-0.011	0.204	-0.007	-0.032	0.015
	ln QChicken$_{t-1}$	0.002	-0.039	0.033	0.014	0.002	-0.021	0.010	0.016	0.004	-0.018	-0.014	-0.007	0.204	-0.033	-0.001
	ln QMilk$_{t-1}$	-0.001	-0.028	0.022	-0.016	0.017	0.013	0.000	-0.012	0.007	0.017	-0.002	-0.032	-0.033	0.204	0.011
	ln QWheat$_{t-1}$	0.000	0.020	-0.016	0.010	-0.005	-0.007	0.007	-0.017	0.004	-0.025	0.029	0.015	-0.001	0.011	0.204
Δ ln PLamb$_t$	α_1	0.282	0.076	0.181	-0.087	-0.037	0.000	0.125	-0.003	0.080	0.000	-0.004	0.043	0.003	-0.001	0.000
	α_2	0.076	0.282	-0.160	-0.038	-0.080	0.058	0.007	-0.101	-0.006	0.005	0.048	0.055	-0.054	-0.039	0.028
	μ_0	0.181	-0.160	0.282	-0.044	0.032	-0.045	0.101	0.078	0.073	-0.004	-0.042	-0.007	0.045	0.030	-0.022
	ln PBeef$_{t-1}$	-0.087	-0.038	-0.044	0.282	-0.045	-0.075	-0.040	0.045	-0.033	0.004	-0.015	0.032	0.019	-0.023	0.014
	ln PPork$_{t-1}$	-0.037	-0.080	0.032	-0.045	0.282	0.028	-0.015	0.011	-0.015	-0.033	0.007	0.022	0.003	0.023	-0.006
	ln PLamb$_{t-1}$	0.000	0.058	-0.045	-0.075	0.028	0.282	-0.028	0.002	-0.051	-0.007	-0.003	0.027	-0.030	0.018	-0.010
	ln PChicken$_{t-1}$	0.125	0.007	0.101	-0.040	-0.015	-0.028	0.282	0.000	0.053	0.012	0.002	-0.011	0.014	0.000	0.010
	ln PMilk$_{t-1}$	-0.003	-0.101	0.078	0.045	0.011	0.002	0.000	0.282	-0.020	0.000	-0.047	-0.011	0.022	-0.017	-0.024
	ln PWheat$_{t-1}$	0.080	-0.006	0.073	-0.033	-0.015	-0.051	0.053	-0.020	0.282	0.003	0.004	-0.004	0.006	0.010	0.006
	ln QCattle$_{t-1}$	0.000	0.005	-0.004	0.004	-0.033	-0.007	0.012	0.000	0.003	0.282	-0.020	-0.133	-0.025	0.024	-0.035
	ln QPig$_{t-1}$	-0.004	0.048	-0.042	-0.015	0.007	-0.003	0.002	-0.047	0.004	-0.020	0.282	-0.015	-0.019	-0.003	0.040
	ln QSheep$_{t-1}$	0.043	0.055	-0.007	0.032	0.022	0.027	-0.011	-0.011	-0.004	-0.133	-0.015	0.282	-0.009	-0.044	0.020

[illegible]
